# Feature fusion and WOA-GWO optimization for Alzheimer’s disease detection with sparse EEG channels

**DOI:** 10.3389/fncom.2026.1835802

**Published:** 2026-05-08

**Authors:** Ruofan Wang, Jitong Wang, Jiaxuan Cai, Siqian Wang, Zixuan Bai, Yanqiu Che

**Affiliations:** 1 School of Information Technology Engineering, Tianjin University of Technology and Education, Tianjin, China; 2 Department of Computational Biology, Imperial College London, London, United Kingdom; 3 Faculty of Medical Technology, Tianjin Medical University, Tianjin, China; 4 Tianjin Key Laboratory of Information Sensing and Intelligent Control, School of Automation and Electrical Engineering, Tianjin University of Technology and Education, Tianjin, China

**Keywords:** Alzheimer’s disease, channel selection, electroencephalogram, feature fusion, nonlinear dynamics, WOA-GWO hybrid optimization

## Abstract

Alzheimer’s Disease (AD) is a neurodegenerative disorder with insidious onset, making early diagnosis challenging. Electroencephalogram (EEG) is a promising noninvasive tool for AD diagnosis, but high-density EEG configurations cause computational burdens and hinder clinical translation. Thus, developing an efficient sparse EEG channel selection method with high classification accuracy is urgent for AD auxiliary diagnosis. This study proposes a multi-strategy enhanced Whale Optimization Algorithm-Grey Wolf Optimizer (WOA-GWO) hybrid model for EEG channel selection, combined with a nonlinear dynamic feature fusion framework. We extracted geometric features from second-order difference plot (SODP) and complexity features (sample entropy, fuzzy entropy) of EEG signals, then adopted the ReliefF algorithm for feature fusion and key feature selection. The WOA-GWO model was improved via chaotic initialization, nonlinear convergence factors, spiral-hierarchical position update, and random perturbation to avoid local optima. Experimental results show that the proposed framework achieves a classification accuracy of 96.97% for AD detection, with significantly reduced EEG channel dimensions (four optimal channels identified: T5, FP1, T4, F4). The WOA-GWO model outperforms the original WOA and GWO in convergence speed and optimization accuracy, and the fused features exhibit strong discriminability for AD-related EEG abnormalities. This work provides a reliable computational framework for developing lightweight, portable AD diagnostic systems, and the identified optimal EEG channels offer neurophysiological evidence for AD electrophysiological biomarkers.

## 1 Introduction

Alzheimer’s Disease (AD) is a brain disorder characterized by neurodegenerative changes, with its core pathological mechanism involving the progressive destruction of neurons and their synaptic connections, ultimately leading to memory impairment, cognitive dysfunction, and behavioral disturbances. As a highly devastating neurological disease, AD not only significantly compromises patients’ ability to perform daily activities but also gradually erodes their capacity for independent living, eventually resulting in death due to multiple organ failure or other complications. As the predominant form of dementia, AD accounts for over 80% of global dementia cases and represents one of the leading causes of disability and mortality among the elderly population. When COVID-19 entered the list of top 10 causes of death, AD ranked as the seventh leading cause of death in the United States and the fifth leading cause of death among Americans aged 65 and older (Better, 2023). During the period from 2000 to 2021, while mortality rates from stroke, heart disease, and HIV declined, reported mortality from Alzheimer’s disease increased by more than 140%. According to the latest statistics, approximately 42.3 million people worldwide are currently living with Alzheimer’s disease, and this number is projected to exceed 152 million by 2050, posing substantial challenges to global healthcare systems and social resources (Catalá-López and Collaborators, 2022; Miltiadous et al., 2023a).

Electroencephalogram (EEG), serving as a noninvasive, cost-effective, readily accessible neurophysiological tool with high temporal resolution, has been extensively employed for detecting cortical electrical activity associated with Alzheimer’s disease (Chetty et al., 2024). Recent investigations have demonstrated that AD progression is characterized by EEG slowing, reduced signal complexity, and disrupted neural synchrony (Akbar et al., 2025; Lee et al., 2025). The acquisition of EEG data has witnessed a continuous increase in the number of deployed electrodes, including configurations of 32, 64, 128 channels, and beyond. While such high-density montages enhance the spatial resolution of EEG recordings, they simultaneously introduce substantial computational challenges in processing massive datasets, thereby impeding rapid identification of disease progression (Lee et al., 2025). In response to the escalating channel counts in EEG configurations, there exists an urgent imperative to develop methodologies capable of effectively selecting necessary and sufficient electrode subsets for expedited patient assessment.

In recent years, significant advancements have been achieved in EEG signal analysis methodologies, encompassing entropy-based features, power spectral density, wavelet transform, complex network analysis, event-related potentials, and second-order difference plots (SODP), among others. The SODP represents an analytical tool grounded in second-order signal differencing, primarily designed for extracting geometric characteristics from temporal series data. Its technical principle involves computing the difference between the current signal value and its preceding two temporal values, thereby accentuating local signal variations. This differential approach endows SODP with exceptional performance in processing nonlinear and nonstationary signals, effectively reducing data dimensionality while providing intuitive visualization capabilities for complex signal analysis. Sun and Chen (2023) applied SODP to electrocardiogram (ECG) signal analysis for the diagnosis of congestive heart failure (CHF). By partitioning SODP into quadrant regions of varying radii and calculating point distributions within each quadrant to extract feature vectors, in conjunction with neural network classifiers, they achieved 100% accuracy in discriminating between normal subjects and CHF patients. Akbari et al. (2021) extracted geometric features from SODP of EEG signals and employed the Binary Particle Swarm Optimization (BPSO) algorithm to reduce feature dimensionality from 26 to 12 or 13 features. This methodology attained an average classification accuracy of 98.79% under 10-fold cross-validation, demonstrating its high efficacy and precision in depression detection.

Although the SODP analysis enables the elucidation of dynamic characteristics of EEG signals from multiple perspectives, thereby providing a comprehensive viewpoint for understanding neurological states and their alterations, several challenges persist in its feature extraction procedure. Specifically, the substantial increase in extracted feature quantities attributable to inter-feature correlations may exert deleterious effects upon model classification performance. For instance, if *n* features are extracted from a single channel and *x* channels are employed in total, the aggregate feature dimensionality will amount to *n* × *x*. Under such circumstances, feature fusion emerges as an efficacious strategy for addressing multi-channel and multi-feature complexities. Concretely, features extracted from each individual channel are aggregated into a singular representative feature that encapsulates the comprehensive characteristics of that channel. Subsequently, the application of intelligent algorithms for feature selection upon these fused representations enables effective management of scenarios involving substantial numbers of channels and features.

The ReliefF algorithm demonstrates superior performance in the feature dimensionality reduction and selection process. Originally proposed by Kira and Rendell in 1992 for binary classification tasks (Urbanowicz et al., 2018), the algorithm was subsequently extended to ReliefF by Kononenko in 1994 to address more complex scenarios involving multi-class classification, noisy data, and missing values (Kononenko, 1994). The superiority of ReliefF over other traditional feature fusion and selection methods (such as statistical-based or PCA) has been extensively validated in recent literature. For instance, in epilepsy seizure data classification studies, the integration of ReliefF with linear Support Vector Machines (SVM) for feature screening and classification has significantly enhanced classification accuracy (Atlam et al., 2025). In the field of image processing, ReliefF has been utilized to select critical features from extracted feature descriptors, thereby improving the performance of fault image recognition (Yang et al., 2025). In data mining applications, the algorithm is frequently applied to handle imbalanced data for classification optimization (Rojarath et al., 2025). Our previous studies have verified the effectiveness of the ReliefF algorithm in feature fusion (Wang et al., 2024, 2026). These applications collectively demonstrate that ReliefF maintains an excellent balance between computational efficiency and feature redundancy reduction. Nevertheless, the application of ReliefF for feature fusion in the early detection of Alzheimer’s disease based on EEG signals remains in the initial stage. Currently, the Whale Optimization Algorithm-Grey Wolf Optimizer (WOA-GWO) hybrid optimization algorithm has demonstrated exceptional performance across diverse engineering and scientific research domains, attributable to its remarkable capability in balancing exploration and exploitation. For instance, in global numerical optimization problems such as pressure vessel design, this algorithm has proven its superior search efficiency (Mohammed and Rashid, 2020). In energy and power systems, it has been successfully applied to multi-objective optimization of cost and water consumption in multi-area dynamic economic dispatch (Ghanbari and Lotfi, 2025). In complex scenarios including epidemic prediction (Alizadeh et al., 2026), Internet of Healthcare Things (IoHT) attack detection (Akash et al., 2025), and Network-on-Chip (NoC) test scheduling (Chandrasekaran et al., 2025), WOA-GWO consistently exhibits enhanced accuracy and stability compared to standalone heuristic algorithms. Specifically, technical solutions that integrate ReliefF’s filter-based selection capability with metaheuristic optimization algorithms to achieve high-precision channel selection and feature dimensionality reduction remain scarcely documented in the existing literature. This study aims to address this research gap by constructing a hybrid optimization architecture, thereby enhancing the sensitivity and specificity of AD detection.

This study investigated EEG signal abnormalities in Alzheimer’s disease patients, extracting 12 geometric and entropy features based on the SODP for AD detection and analysis. Via Maximum Cross-Correlation (MCC)-based correlation visualization, eight features with minimal inter-feature correlations were selected, followed by statistical screening and stability validation. The ReliefF algorithm was then applied to fuse the most representative features, yielding a single fused feature vector for each EEG channel. Finally, a hybrid WOA-GWO was adopted for channel selection, which outperforms the original Whale Optimization Algorithm (WOA) and Grey Wolf Optimizer (GWO) with notable optimization advantages. This hybrid algorithm enhances the diversity and spatial coverage of initial solutions via chaotic mapping-based population initialization, and dynamically adjusts search steps through a nonlinear convergence factor to balance global exploration and local exploitation adaptively. In the position update phase, it integrates the spiral predation mechanism of WOA with the hierarchical leadership of GWO, adopting a weighted adaptive strategy to coordinate the co-evolution of the two populations; periodic random perturbations are also introduced to strengthen local optima avoidance. This hybrid mechanism effectively mitigates the premature convergence of single algorithms, exhibiting superior convergence stability and optimization accuracy on standard benchmark functions, and thus providing a reliable optimization framework for high-dimensional feature selection tasks.

Compared to existing hybrid optimization methods and EEG-based Alzheimer’s disease detection approaches, the integrated detection framework proposed in this study demonstrates significant and outstanding innovation. While various metaheuristic algorithms have been employed for feature selection, they often face limitations such as unstable convergence performance and susceptibility to local optima when processing high-dimensional EEG data. Meanwhile, current AD detection models commonly exhibit excessive reliance on full-electrode configurations, resulting in high computational complexity and insufficient clinical interpretability (Shaikh et al., 2025). To address these challenges, this study introduces, for the first time in the field of AD EEG detection, a multi-strategy enhanced WOA-GWO hybrid optimization algorithm specifically designed for sparse channel selection. By integrating chaotic initialization, nonlinear convergence factors, spiral hierarchical position update mechanisms, and periodic random perturbation strategies, the algorithm effectively mitigates the premature convergence defects inherent in independent WOA and GWO algorithms, significantly improving the accuracy and stability of channel search in high-dimensional spaces. This provides a novel optimization paradigm for EEG channel selection in AD auxiliary diagnosis. Additionally, this study establishes a complete and efficient nonlinear dynamic feature fusion process: first, geometric morphological features derived from SODP and signal complexity features represented by sample entropy and fuzzy entropy are weighted and fused via the ReliefF algorithm (Cataldo et al., 2024), followed by key feature screening to form a highly discriminative feature set. This method comprehensively captures AD-related EEG abnormalities from both geometric trajectory and nonlinear complexity perspectives, a feature combination and hierarchical fusion pattern that has not been fully validated in prior research. Finally, through an optimized screening approach, this study obtains a sparse channel subset comprising only four electrodes. These channels exhibit precise correspondence with core brain regions affected by AD pathological damage, significantly reducing computational burden while maintaining exceptionally high detection accuracy. This achievement provides critical support for realizing lightweight, portable, and clinically translatable AD early screening systems, achieving a notable leading advantage over existing similar detection methods in balancing high precision, interpretability, and lightweight deployment.

The main contributions of this study are summarized as follows:

- A multi-strategy enhanced WOA-GWO hybrid optimization algorithm is proposed for EEG channel selection, which integrates chaotic initialization, nonlinear convergence factors, spiral-hierarchical position update, and random perturbation to balance global exploration and local exploitation, effectively avoiding local optima.
- A multi-level nonlinear dynamic feature engineering system (SODP+entropy feature extraction → statistical validation → ReliefF fusion) is constructed, which captures AD-related EEG abnormalities from both geometric morphology and signal complexity perspectives, improving feature discriminability and reducing dimensionality.
- The neurophysiologically interpretable optimal EEG channels (T5, FP1, T4, F4) for AD detection are identified, and a ternary mapping relationship among EEG electrodes, brain region functions, and AD pathological mechanisms is established, providing evidence for AD electrophysiological biomarkers.

## 2 Data acquisition and preprocessing

### 2.1 Data acquisition

**Figure 1:**
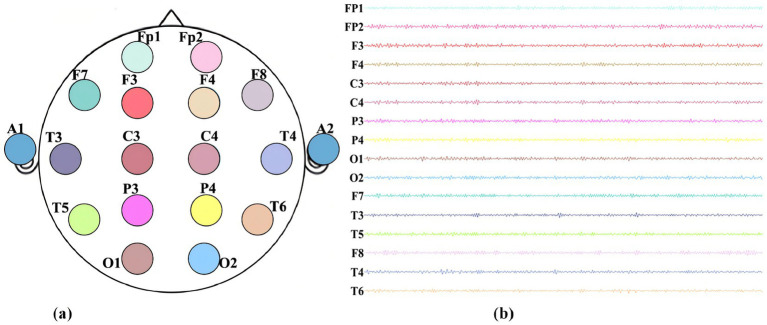
Alzheimer’s disease patient data: **(a)** electrode names and positions, **(b)** 16-channel EEG signals.

This study employed a 16-channel EEG amplifier (Symtop Instrument, Beijing, China) to acquire resting-state EEG signals, with a sampling rate set at 1,024 Hz and a band-pass filter range of 0–60 Hz. Following the international 10–20 electrode placement system, 16 Ag/AgCl electrodes (Fp1, Fp2, F3, F4, C3, C4, P3, P4, O1, O2, F7, F8, T3, T4, T5, and T6) were positioned on the scalp of each subject, as illustrated in Figure 1a, with bilateral earlobes serving as reference electrodes to ensure effective capture of electrical activity across multiple brain regions. A total of 30 right-handed subjects were enrolled, comprising 15 patients with Alzheimer’s disease and 15 age-matched healthy controls (mean age approximately 75 years). All AD patients were diagnosed with dementia according to the criteria established by the World Health Organization’s International Classification of Diseases (ICD-10) and the Diagnostic and Statistical Manual of Mental Disorders (DSM-IV). Rigorous screening was conducted to exclude individuals with comorbid neurological or psychiatric disorders, recent use of neuroactive medications, or other factors potentially confounding EEG activity. The two groups were well-matched in terms of gender distribution (AD group: male:female = 8:7; control group: 9:6) and years of education (>6 years for all subjects). Mini-Mental State Examination (MMSE) scores revealed that the AD group (21.3 ± 5.8) was significantly lower than the control group (27.1 ± 1.3, *p* < 0.001). All subjects completed a 10-min resting-state EEG recording session (eyes closed for the first 5 min and eyes open for the last 5 min) in a quiet, electromagnetically shielded environment. Concurrent structural neuroimaging data, including cranial CT and structural magnetic resonance imaging (MRI), were obtained to assist in clinical evaluation.

This study was approved by the Research Ethics Committee of Tianjin University of Technology and Education (Approval No.: 2024-04). All subjects provided written informed consent prior to participation, having been fully informed of the research objectives, experimental procedures, and potential risks. The entire study was conducted in strict accordance with the ethical principles outlined in the Declaration of Helsinki, ensuring the protection of subjects’ autonomy, privacy, and safety.

### 2.2 EEG data preprocessing

#### 2.2.1 Data screening and epoch selection

The middle segment (2–4 min) of the eyes-closed resting condition was selected as valid data to mitigate the influence of equipment startup transients and initial subject adaptation, ensuring that the EEG signals were in a stable physiological state and reducing non-neurogenic fluctuation interference. The original data were acquired at a sampling rate of 1,024 Hz; following epoch selection, 120 s of continuous EEG recordings were retained for each AD patient for further processing.

#### 2.2.2 Epoch segmentation and sample augmentation

To enhance the diversity of training samples and meet the data volume requirements of machine learning algorithms, researchers sequentially segmented each subject’s 120-s effective electroencephalogram (EEG) recording into 12 non-overlapping 10-s epochs (each containing 10,240 sampling points). This segmentation and partitioning procedure strictly followed the previous standardized protocol (Wang et al., 2024, 2026). Through this segmentation strategy, the original recordings from 15 Alzheimer’s disease (AD) patients were expanded into 180 independent analysis units, effectively mitigating overfitting risks associated with small-sample datasets while preserving intra-individual temporal variability to enhance the robustness of feature representation.

#### 2.2.3 Artifact identification and manual rejection

Offline visual inspection combined with time-frequency analysis was employed to screen segmented epochs for artifacts. Two trained neuroelectrophysiology technicians independently reviewed signals from all channels, with particular attention to identifying and rejecting transient interference caused by eye movements, blinks, muscle activity, and poor electrode contact. Any 10-s segment containing significant artifacts was entirely excluded to ensure that data units incorporated into the analysis possessed favorable signal-to-noise ratios. Although time-consuming, this manual verification procedure prevents the misclassification of subtle pathological signals by automated algorithms, thereby safeguarding data quality. To reduce spurious functional connectivity arising from volume conduction in scalp electrode recordings, this study applied the Surface Laplacian Transform method proposed by Wang et al. to spatially filter the 16-channel raw signals. By estimating the spatial second derivative of scalp surface potentials, this method effectively suppresses far-field interference and enhances the spatial specificity of local neural source activity, thereby improving the physiological validity of subsequent functional network construction. All computations were implemented using spherical spline interpolation, with reference electrodes set to the combined bilateral earlobes.

#### 2.2.4 Data standardization

The entire preprocessing workflow was conducted in MATLAB R2021b, with core operations implemented via the EEGLAB 2021.1 toolbox. All channel signals underwent zero-mean and unit-variance normalization to eliminate the influence of inter-individual amplitude differences on feature extraction. The final dataset was cross-verified by two independent reviewers to ensure the absence of residual artifacts and that the power spectral distribution across all frequency bands conformed to the physiological characteristics of resting-state EEG. Representative preprocessed raw waveforms are presented in Figure 1b, thereby validating the efficacy of the preprocessing pipeline.

## 3 Method

### 3.1 SODP

The complexity of EEG signals can be quantified through the two-dimensional spatial patterns of second-order difference plots (SODPs) (Akbari et al., 2021). In SODP, x (n + 2) − x (n + 1) is plotted against x (n + 1) − x (n), where x (n) represents the value at discrete time n, reflecting the correlation between consecutive values in the time series. By plotting the relationship between x (n) and y (n), the SODP of EEG signals can be obtained. Specifically, assuming the EEG signal is denoted as EEG(n), x(n) and y(n) are defined as follows (Equations 1, 2):


x(n)=EEG(n+1)−EEG(n) (1)

y(n)=EEG(n+2)−EEG(n+1) (2)

Ultimately, the SODP of the EEG signal can be obtained by plotting the relationship between x(n) and y(n).

#### 3.1.1 Standard descriptors (STD)

To quantify the dispersion of EEG data on the SODP, standard descriptors (STD) were employed as metrics (Akbari et al., 2021). Specifically, STD1 represents the standard deviation of the SODP projection onto the line perpendicular to the identity line (y = x), namely y = −x, whereas STD2 denotes the standard deviation of the SODP projection onto the identity line (y = x). The computational formulas are defined as follows (Equations 3–5):


$$\mathrm{STD}1=\sqrt{\mathrm{Var}(\frac{x(n)-y(n)}{2^{\frac{1}{2}}})}$$ (3)

$$\mathrm{STD}2=\sqrt{\mathrm{Var}(\frac{x(n)+y(n)}{2^{\frac{1}{2}}})}$$ (4)

STD=π(STD1×STD2) (5)

Where Var(·) denotes variance. In this manner, STD1 and STD2 effectively characterize the distribution features of EEG data on the SODP, providing an important foundation for subsequent analysis.

#### 3.1.2 Summation of the angles between consecutive vectors (SAV)

The summation of the angles between consecutive vectors (SAV) was employed as a geometric feature to quantify the directional variation of adjacent vectors on the SODP, serving as a measure of EEG signal complexity in the temporal domain (Akbari et al., 2021). This metric not only reveals the dynamic interaction characteristics among different EEG channels but also effectively captures subtle structural changes in signal evolution over time. The calculation of SAV is based on the angle between two vectors formed by three consecutive points on the SODP, with the formula defined as follows (Equations 6–8):


$$a=(x_{i+1}-x_{i},y_{i+1}-y_{i})=(x_{m},y_{m})$$ (6)

$$b=(x_{i+2}-x_{i+1},y_{i+2}-y_{i+1})=(x_{m+1},y_{m+1})$$ (7)

$$\mathrm{SAV}=\sum_{m=1}^{n-2}\frac{x_{m}x_{m+1}+y_{m}y_{m+1}}{\sqrt{x_{m}^{2}+y_{m}^{2}}+\sqrt{x_{m+1}^{2}+y_{m+1}^{2}}}$$ (8)

#### 3.1.3 Sum of distances to coordinate (SDC)

In the SODP representation, data points of normal EEG signals typically exhibit more extensive distribution characteristics compared to abnormal signals, with significantly increased distances from the coordinate origin (Wang et al., 2022). This geometric property is utilized to extract features such as the Sum of Distances to Coordinate (SDC) to quantify spatial distribution differences between signals. By accumulating the distances from each point in the SODP to the coordinate axes, an effective metric for distinguishing normal from abnormal EEG signals is obtained. The calculation results are as follows (Equation 9):


$$\mathrm{SDC}=\sum_{i=1}^{n-2}\sqrt{x{\left(i\right)}^{2}+y{\left(i\right)}^{2}}$$ (9)

#### 3.1.4 Sum of the triangle area using consecutive vectors (STA)

The angles formed between consecutive vectors constitute multiple triangular structures. When the areas of these triangles and their interior angles tend toward smaller values, it indicates a certain degree of attenuation in the dynamic characteristics of the system (Xie et al., 2017). To quantify this variation, the sum of triangular areas formed by consecutive vectors can be employed as a feature index, namely the STA. The formula is presented as follows (Equation 10):


$$\mathrm{STA}=\frac{1}{2}\sum_{i-1}^{n-2}|\det[\begin{array}{ccc} x_{i} & x_{i+1} & x_{i+2}\\ y_{i} & y_{i+1} & y_{i+2}\\ 1 & 1 & 1 \end{array}]|$$ (10)

#### 3.1.5 Sum of the shortest distance of each point from the 45-degree line (SSHD)

To quantify the broader geometric characteristics of normal EEG signals in the SODP compared to EEG signals of AD patients, this study employed a method calculating the shortest distance from each data point to the 45-degree line for feature extraction. Specifically, for each point (x_i, y_i) on the SODP, the shortest distance to the line y = x was computed, and the sum of all such shortest distances was accumulated as the final quantitative index. This metric is termed the “Sum of Shortest distances to the 45-degree line (SSHD),” with its computational formula defined as follows (Equation 11):


$$\text{SSHD}=\sum_{i=1}^{n-2}\frac{|x(i)-y(i)|}{\sqrt{2}}$$ (11)

Where n represents the total number of data points in the SODP. A larger SSHD value indicates more dispersed distribution of the signal in the two-dimensional space, reflecting stronger system dynamics; conversely, a smaller SSHD value signifies attenuated signal dynamics.

#### 3.1.6 Summation of the centroid to centroid distance of successive triangles (SCC)

To quantify the self-similarity of SODP patterns, this study introduced the distance between centroids of consecutive triangles as a geometric feature. Specifically, triangles were first constructed from every three consecutive points on the SODP, and their centroid coordinates were calculated (Equations 12, 13):


$$x_{C}=\frac{x_{i}+x_{i-1}+x_{i-2}}{3}$$ (12)

$$y_{C}=\frac{y_{i}+y_{i-1}+y_{i-2}}{3}$$ (13)

The Euclidean distance between the centroids of adjacent triangles was then computed, and the sum of all adjacent centroid distances was accumulated to obtain the feature value SCC (Equation 14):


$$\mathrm{SCC}=\sum \sqrt{{\left(x_{C_{i+1}}-x_{C_{i}}\right)}^{2}+{\left(y_{C_{i+1}}-y_{C_{i}}\right)}^{2}}$$ (14)

This feature effectively reflects the structural repetitiveness and local variation trends of SODP patterns, serving as an important indicator for measuring signal self-similarity.

#### 3.1.7 Sum successive of vectors length (SSVL)

In SODP analysis, the mapping of vectors between consecutive points is employed to reflect amplitude variations of EEG signals in the temporal domain. The total length of consecutive vectors was adopted as a feature to quantify the coverage distance of EEG signals on the SODP coordinate plane. The specific computational formula is as follows (Equation 15):


$$\text{SSVL}=\sum_{i=1}^{n-1}\sqrt{{\left(x_{i+1}-x_{i}\right)}^{2}+{\left(y_{i+1}-y_{i}\right)}^{2}}$$ (15)

#### 3.1.8 Central tendency measure (CTM)

The Center Tendency Measure (CTM) was utilized to evaluate the dispersion degree of EEG signals in the SODP plot. The CTM value reflects the number of data points contained within the SODP plot; a larger CTM value indicates broader distribution range and greater variability of the signal in phase space. The definition of CTM is as follows (Equations 16, 17):


$$\mathrm{CTM}=\frac{1}{N}\sum_{n=1}^{N}q(b_{n})$$ (16)

$$q(b_{n})=\{\begin{array}{c} 1,\text{if}\sqrt{{\left[x(n)\right]}^{2}+{\left[y(n)\right]}^{2}}\leq r\\ 0,\text{otherwise} \end{array}$$ (17)

These eight SODP geometric features capture the spatial and temporal dynamic characteristics of EEG signals, and combining them with entropy features can further reflect the complexity of AD-related EEG abnormalities, thus forming a comprehensive nonlinear dynamic feature set.

### 3.2 Entropy features (FE)

Entropy is an important metric for evaluating the rate of information generation and is widely applied in the analysis of EEG signals. It can effectively distinguish useful signals from background noise (Acharya et al., 2012). Typically, higher entropy values indicate greater irregularity or unpredictability of signals, whereas lower entropy values suggest higher regularity. In this study, sample entropy and hybrid entropy were employed as characteristic parameters to characterize and analyze the complexity and irregularity of EEG signals in patients with Alzheimer’s disease.

#### 3.2.1 Sample entropy (SE)

Sample entropy is an information-theoretic statistic used to measure the irregularity or complexity of time series data. This method demonstrates strong robustness against short-duration intense transient interference (such as spike noise) while quantifying signal complexity. Consequently, sample entropy has been extensively applied in nonlinear analysis of physiological signals (Ahmadieh and Ghassemi, 2022). In this study, sample entropy was selected as a feature parameter for analyzing EEG signals in AD patients precisely based on these aforementioned advantages (Equations 18–21).

Given a time series of length N, denoted as $\left\{x_{1},,,x_{2},,,\ldots ,,,x_{N}\right\}$ , m-dimensional phase space vectors are first constructed:


$$X_{i}^{m}=[x_{i},,,x_{i+1},,,\ldots ,,,x_{i+m-1}],\;\;i=1,2,\ldots ,N-m+1$$ (18)

The Chebyshev distance between two vectors is defined as:


$$d[X_{i}^{m},X_{j}^{m}]=\max_{0\leq k\leq m-1}|x_{i+k}-x_{j+k}|,\;\;j\neq i$$ (19)

Under the tolerance threshold r (typically set as r = 0.15 × SD, where SD represents the standard deviation of the sequence), the proportion of vector pairs satisfying $d[X_{i}^{m},X_{j}^{m}]\leq r$ is calculated:


$$B^{m}(r)=\frac{1}{N-m}\sum_{i=1}^{N-m}\frac{1}{N-m-1}\;\;\sum_{\begin{array}{c} j=1\\ j\neq i \end{array}}^{N-m}\Theta (r-d[X_{i}^{m},X_{j}^{m}])$$ (20)

Where Θ(·) denotes the Heaviside step function.

Similarly, the matching proportion $B^{m+1}(r)$ for (m + 1)-dimensional vectors is computed. Sample entropy is defined as:


$$\text{SampEn}(m,r,N)=-\ln[\frac{B^{m+1}(r)}{B^{m}(r)}]$$ (21)

In this study, m = 2 and r = 0.15 × SD were adopted as the standard parameter configuration.

#### 3.2.2 Fuzzy entropy (FE)

Fuzzy Entropy is a metric employed to measure signal complexity, offering the advantage of insensitivity to noise interference (Arpaia et al., 2024). It has been extensively applied in EEG signal analysis, with higher values indicating more complex dynamic variations in the time series. The computational procedure of fuzzy entropy is as follows (Equations 22–26):

Given a time series of length N, denoted as ${\left\{x(i)\right\}}_{i=1}^{N}$ , phase space reconstruction is first performed to generate m-dimensional vectors:


$$\begin{array}{l} X_{i}^{m}=[x(i),x(i+\tau ),\ldots ,x(i+(m-1)\tau )],\\ \;\;\;\;\;\;\;\;\;\;i=1,2,\ldots ,N-(m-1)\tau \end{array}$$ (22)

Where m represents the embedding dimension and τ denotes the time delay.

The Chebyshev distance between two vectors $X_{i}^{m}$ and $X_{j}^{m}$ ( j≠i ) is defined as:


$$d_{\mathrm{ij}}^{m}=\max_{0\leq k\leq m-1}|x(i+\mathrm{k\tau})-x(j+\mathrm{k\tau})|$$ (23)

A fuzzy function is introduced to map the distance to the interval [0, 1]:


$$\mu (d_{\mathrm{ij}}^{m},n,r)=\exp[-{\left(\frac{d_{\mathrm{ij}}^{m}}{r}\right)}^{n}]$$ (24)

Where r is the tolerance threshold (typically set as r = 0.15 × SD, where SD represents the standard deviation of the sequence), and n is the fuzzy exponent (typically set as n = 2).

The matching probability for m-dimensional vectors is calculated:


$$\phi^{m}(n,r)=\frac{1}{N-\mathrm{m\tau}}\sum_{i=1}^{N-\mathrm{m\tau}}[\frac{1}{N-(m+1)\tau }\sum_{\begin{array}{c} j=1\\ j\neq i \end{array}}^{N-\mathrm{m\tau}}\mu (d_{\mathrm{ij}}^{m},n,r)]$$ (25)

Similarly, the matching probability $\phi^{m+1}(n,r)$ for (m + 1)-dimensional vectors is computed. Fuzzy entropy is defined as:


$$\text{FuzzyEn}(m,,,n,,,r,,,N)=\ln\phi^{m}(n,r)-\ln\phi^{m+1}(n,r)$$ (26)

In this study, the standard parameter configuration was adopted: m = 2, τ = 1, n = 2, and r = 0.15 × SD. Compared with sample entropy, fuzzy entropy replaces the step function with an exponential membership function, rendering the distance metric continuous and differentiable, thereby demonstrating enhanced robustness to parameter selection and noise interference.

Sample entropy and fuzzy entropy were selected for their strong robustness to noise and high sensitivity to AD-related EEG complexity changes, and the fusion of these entropy features with SODP geometric features can improve the discriminability of EEG features for AD detection.

### 3.3 Whale optimization algorithm (WOA)

The Whale Optimization Algorithm, first proposed by Australian scholars Mirjalili and Lewis in 2016, is a novel swarm intelligence optimization method inspired by the predatory behavior of humpback whales (Mirjalili and Lewis, 2016; Chakraborty et al., 2023; Nadimi-Shahraki et al., 2023). Through mathematical modeling, this algorithm simulates three typical behaviors of whale pods during the “bubble-net attacking” process: encircling prey, spiral updating, and random searching, to solve continuous optimization problems. After identifying the prey position, whales update their individual positions according to the following (Equations 27–32):


$$\vec{D}=|\vec{C}\cdot {\vec{X}}^{*}(t)-\vec{X}(t)|$$ (27)

$$\vec{X}(t+1)={\vec{X}}^{*}(t)-\vec{A}\cdot \vec{D}$$ (28)

Where ${\vec{X}}^{*}(t)$ denotes the current optimal solution vector, $\vec{X}(t)$ represents the current whale position vector, and coefficients $\vec{A}$ 和 $\vec{C}$ are calculated as follows:


$$\vec{A}=2\vec{a}\cdot {\vec{r}}_{1}-\vec{a},\;\;\vec{C}=2\cdot {\vec{r}}_{2}$$ (29)

$\vec{a}$ linearly decreases from 2 to 0 with iterations; ${\vec{r}}_{1},{\vec{r}}_{2}$ ∈[0,1] are uniform random vectors.

During the bubble-net attacking phase, two strategies are considered simultaneously, with one selected at a probability of 50%:

a Shrinking Encircling Mechanism

This is implemented through the equation $\vec{X}(t+1)={\vec{X}}^{*}(t)-\vec{A}\cdot \vec{D}$ , achieved by setting ∣ $\vec{A}$ ∣ < 1.

b Spiral Updating Position

Whales swim toward the prey along a spiral path:


$$\vec{X}(t+1)={\vec{D}}^{\prime }\cdot e^{\mathrm{bl}}\cdot \cos(2\mathrm{\pi l})+{\vec{X}}^{*}(t)$$ (30)

${\vec{D}}^{\prime }=|{\vec{X}}^{*}(t)-\vec{X}(t)|$ represents the distance between the whale and the prey; constant b defines the logarithmic spiral shape, and *l* ∈ [−1,1] is a random number.

Search for Prey Phase, when ∣ $\vec{A}$ ∣ ≥ 1 whales abandon the current optimal solution and randomly select another whale as reference:


$$\vec{D}=|\vec{C}\cdot {\vec{X}}_{\mathrm{rand}}-\vec{X}|$$ (31)

$$\vec{X}(t+1)={\vec{X}}_{\mathrm{rand}}-\vec{A}\cdot \vec{D}$$ (32)

This mechanism enhances global exploration capability and prevents premature convergence.

### 3.4 Grey wolf optimizer (GWO)

The Grey Wolf Optimizer, proposed by Australian scholar Mirjalili et al. (2014), is a novel swarm intelligence optimization algorithm that simulates the social hierarchy and cooperative hunting behavior of grey wolf packs. GWO abstracts the hunting process into three stages—“encircling, pursuing, and attacking”—through the role division of three leader wolves (*α*, *β*, *δ*) and ordinary wolves (*ω*), thereby completing the search process from global exploration to local exploitation. The detailed mechanism of GWO is summarized in Table 1. Since its inception, the Grey Wolf Optimizer has been widely applied in power system optimization (Hosseini-Hemati et al., 2022), wireless sensor network node deployment, and automatic hyperparameter tuning of deep learning (Shokouhandeh et al., 2021), demonstrating superior optimization performance and robustness compared to traditional methods such as Particle Swarm Optimization (PSO) and Genetic Algorithm (GA) (Makhadmeh et al., 2023).

**Table 1:** Grey wolf hierarchy and algorithm roles.

Grey wolf hierarchy	Algorithm role	Mathematical meaning
α (Leader)	Current optimal solution	Individual with best fitness
β (Sub-leader)	Second-best solution	Individual with second-best fitness
δ (Executor)	Third-best solution	Individual with third-best fitness
ω (Ordinary wolf)	Remaining individuals	Update positions based on the top three

In the encircling prey phase, let the prey position be X_p_ and the position of the i-th grey wolf be X_i_, then the encircling behavior can be expressed as (Equations 33–42):


$$D_{i}=|C⊙X_{p}(t)-X_{i}(t)|$$ (33)

$$X_{i}(t+1)=X_{p}(t)-A⊙D_{i}$$ (34)

Where,


$$A=2ar_{1}-a$$ (35)

$$C=2r_{2}$$ (36)

linearly decreases from 2 to 0 with iteration count t:


$$a=2-t\frac{2}{T_{\max}}$$ (37)

$$r_{1},r_{2}\in [0,1]$$ (38)

$r_{1},r_{2}$ are random vectors, and ⊙ denotes element-wise multiplication.

In the pursuing prey phase, *α*, *β*, and *δ* jointly guide the wolf pack to approach the prey. For any ω wolf, the distances to the three leader wolves are calculated and candidate positions are generated:


$$D_{\alpha }=|C_{1}⊙X_{\alpha }-X|,\;\;X_{1}=X_{\alpha }-A_{1}⊙D_{\alpha }$$ (39)

$$D_{\beta }=|C_{2}⊙X_{\beta }-X|,\;\;X_{2}=X_{\beta }-A_{2}⊙D_{\beta }$$ (40)

$$D_{\delta }=|C_{3}⊙X_{\delta }-X|,\;\;X_{3}=X_{\delta }-A_{3}⊙D_{\delta }$$ (41)

The final position is updated as the average of the three:


$$X(t+1)=\frac{X_{1}+X_{2}+X_{3}}{3}$$ (42)

In the attacking prey phase, when |A| < 1, the wolf pack converges toward the prey (exploitation); when |A| > 1, the wolf pack disperses away from the prey for global search (exploration). The smooth transition from exploration to exploitation is achieved through the decreasing of *a*.

### 3.5 Improved hybrid whale-grey wolf optimization algorithm

To select the optimal sparse EEG channels with high discriminability, a multi-strategy enhanced WOA-GWO hybrid optimization algorithm was proposed, which overcomes the limitations of the original WOA and GWO in high-dimensional feature space. The WOA and GWO are both high-performance swarm intelligence optimization paradigms proposed in recent years. WOA achieves a favorable balance between global exploration and local exploitation by simulating the spiral bubble-net predatory behavior of humpback whales; GWO demonstrates strong convergence accuracy and stability through the strict hierarchical hunting mechanism of grey wolf packs. Both algorithms have been successfully applied in various domains, including feature selection, scheduling, and power system optimization. However, as the dimensionality of optimization problems increases and objective functions exhibit enhanced nonlinearity and multimodal characteristics, both WOA and GWO reveal common deficiencies such as susceptibility to local optima and premature convergence. To overcome these bottlenecks, this study proposes the Hybrid Whale-Grey Wolf Optimization Algorithm, which preserves the core search mechanisms of both algorithms while achieving complementary advantages and deep integration, with the aim of attaining superior global exploration capability and local exploitation precision in complex optimization environments.

WOA-GWO follows a hybrid strategy of “collaborative search, dynamic switching, and information complementarity.” The algorithm divides the search population into two functionally complementary subpopulations:

(1) Whale subpopulation (WOA-subpopulation): Responsible for global exploration, utilizing WOA’s distinctive spiral updating and shrinking encircling mechanisms to rapidly locate potentially high-quality regions in high-dimensional space;
(2) Grey wolf subpopulation (GWO-subpopulation): Responsible for fine-grained exploitation, leveraging GWO’s hierarchical leadership (*α*, *β*, *δ*) strategy to conduct in-depth mining of high-quality regions discovered by the whale subpopulation.

**Figure 2:**
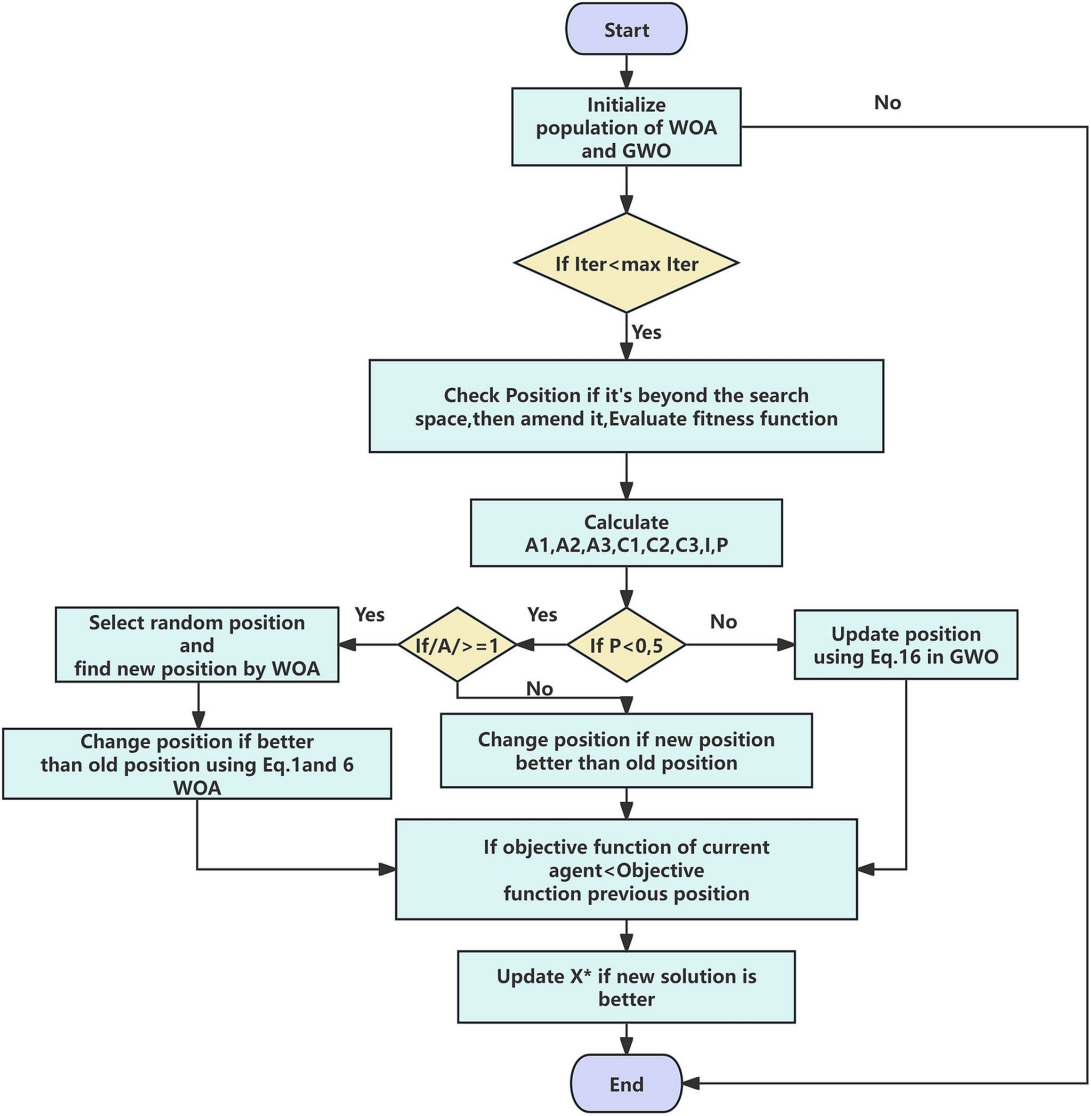
Flowchart of the WOA-GWO algorithm.

The sizes of the two subpopulations are adaptively adjusted throughout the iterative process: the whale subpopulation dominates in early iterations to ensure global exploration, while the proportion of the grey wolf subpopulation increases in later stages to strengthen local convergence. Collaborative synergy between subpopulations is achieved through an elite information sharing mechanism: after each generation, both sides exchange their best individuals (α wolf and best whale), and the global optimum is updated based on fitness ranking, thereby preventing any single algorithm from falling into local extrema. The algorithm flowchart is illustrated in Figure 2.

The WOA-GWO hybrid optimization algorithm employed in this study integrates the advantages of two metaheuristic algorithms through a dynamic collaborative mechanism:

Step 1: Randomly generate a hybrid population comprising N individuals, where the first half adopts the initialization strategy of the WOA and the second half adopts the initialization method of the GWO. Calculate the fitness values of all individuals and determine the current global optimal solution.

Step 2: Perform boundary checking for each individual’s position vector: if any dimension exceeds the preset search space range, apply the boundary reflection strategy to correct the position and recalculate the fitness value. This step ensures that the population always evolves within the valid solution space.

Step 3: Based on the current iteration progress, calculate key control parameters including the convergence factor, random coefficients, and linear weight coefficients, which are used to dynamically regulate the search behavior of the algorithm.

Step 4: Dynamically select the update mechanism according to the random probability P and the threshold of convergence factor |A|: when *p* < 0.5, adopt GWO’s hierarchical leadership mechanism; when |A| ≥ 1, adopt WOA’s random search mechanism; when *p* ≥ 0.5 and |A| < 1, adopt WOA’s spiral predation mechanism. The three mechanisms are adaptively switched according to the iteration progress, ensuring optimal performance of the algorithm at different stages.

Step 5: Evaluate the fitness value of the new position and replace the original position if superior. Simultaneously, regularly compare the fitness values of all individuals and update the global optimal solution X*, ensuring that high-quality solution information is not lost.

Step 6: Determine whether the current iteration count has reached the preset maximum: if not, return to Step 2 to continue iteration; if reached, terminate the algorithm and output the global optimal solution. This process effectively avoids the premature convergence problem of single algorithms through dynamically balancing exploration and exploitation capabilities.

#### 3.5.1 Core improvement strategies of WOA-GWO

In the spiral-encircling collaborative search phase, WOA-GWO couples WOA’s spiral equation with GWO’s encircling equation to form a novel position update rule (Equations 43–45):


$$\vec{X}(t+1)=\frac{{\vec{X}}_{\text{best}}(t)+{\vec{X}}_{\alpha }(t)}{2}+|\vec{C}\cdot {\vec{X}}_{\text{best}}(t)-\vec{X}(t)|\cdot e^{\mathrm{bl}}\cdot \cos(2\mathrm{\pi l})$$ (43)

Where ${\vec{X}}_{\text{best}}$ represents the global optimum of the whale subpopulation, ${\vec{X}}_{\alpha }$ denotes the α wolf position of the grey wolf subpopulation, b and l are spiral shape parameters, and $\vec{C}$ is a random coefficient vector. This equation preserves the spiral characteristic of WOA while introducing α wolf guidance to enhance directionality.

In traditional GWO, the weights of α, *β*, and *δ* wolves are fixed. WOA-GWO introduces adaptive weight factors:


$$w_{i}=\frac{f_{\text{worst}}-f_{i}}{f_{\text{worst}}-f_{\text{best}}},i\in \{\alpha ,\beta ,\delta \}$$ (44)

Where f denotes the fitness value. The weights are dynamically adjusted with iterations, making the algorithm more reliant on *α* wolf guidance in the early search stage while strengthening population diversity in later stages.

To balance exploration and exploitation, WOA-GWO employs a nonlinear convergence factor:


$$a(t)=2-2\cdot \frac{t}{T}\cdot (1-\frac{t}{T})$$ (45)

Where t is the current iteration and T is the maximum iteration. This factor maintains a relatively large value during the middle iterations, prolonging global search time and effectively suppressing premature convergence.

To achieve an adaptive balance between global exploration and local exploitation, and effectively mitigate the premature convergence issue of single metaheuristic algorithms, the proposed WOA-GWO hybrid algorithm incorporates four key optimized strategies for population initialization and position update:

Chaotic initialization: Chaotic mapping is adopted for population initialization to enhance the diversity and spatial coverage of initial solutions, overcoming the random initialization limitation of the original WOA and GWO.

Nonlinear convergence factor: A nonlinear convergence factor is introduced to dynamically adjust the search step size, balancing global exploration and local exploitation throughout the iteration process, and suppressing premature convergence.

Spiral-hierarchical position update: The spiral predation behavior of WOA is integrated with the hierarchical leadership mechanism (α/*β*/*δ*) of GWO to form a weighted adaptive position update rule, improving the directionality and accuracy of the search.

Random perturbation strategy: A periodic random perturbation strategy is introduced to strengthen the ability to escape local optima, which is a critical improvement for high-dimensional EEG channel selection.

### 3.6 CatBoost

The CatBoost algorithm was proposed by Prokhorenkova et al. in CatBoost: unbiased boosting with categorical features (Prokhorenkova et al., 2018). It is based on the gradient boosting framework and employs K additive trees to approximate the output ${\hat{y}}_{i}$ , as illustrated by the following formula (Equations 46–49):


$${\hat{y}}_{i}=\sum_{k=1}^{K}f_{k}(x_{i}),\;\;f_{k}\in F$$ (46)

Where $f_{k}$ represents the k-th classification and regression tree (CART), and F denotes the space of all possible trees.

The objective function of CatBoost consists of a loss function and a regularization term, which can be expressed as:


$$\mathrm{Obj}=\sum_{i=1}^{n}L(y_{i},{\hat{y}}_{i})+\sum_{k=1}^{K}\Omega (f_{k})$$ (47)

Where $L(y_{i},{\hat{y}}_{i})$ denotes the loss for binary classification, and $\Omega (f_{k})$ controls the complexity of each tree to avoid overfitting. The regularization term is defined as:


$$\Omega (f_{k})=\mathrm{\gamma T}+\frac{1}{2}\lambda \sum_{j=1}^{T}w_{j}^{2}$$ (48)

Where T is the number of leaf nodes, *w_j_* is the weight of the j-th leaf, and γ, λ are hyperparameters that constrain model complexity.

CatBoost further introduces ordered boosting and unbiased categorical encoding to reduce prediction shift and improve generalization. The optimal weight of each leaf node is obtained by second-order approximation:


$$w_{j}^{*}=-\frac{G_{j}}{H_{j}+\lambda }$$ (49)

Where $G_{j}$ and $H_{j}$ represent the sum of first-order and second-order gradients on the j-th leaf, respectively.

## 4 Results

### 4.1 Simulation and verification experiments

To systematically evaluate algorithm performance, this study carefully selects 12 highly representative benchmark functions from the 23 functions available in the CEC2023 test suite for empirical validation. The selected functions span three principal categories-unimodal (4 functions), multimodal (4 functions), and hybrid/composite (4 functions)-comprehensively covering essential optimization characteristics including convexity/non-convexity, continuity/discreteness, and constrained/unconstrained properties. Specifically, the unimodal functions—Sphere (f1), Step (f2), Rosenbrock (f4), and Quadratic (f10)-serve to assess the algorithm’s fundamental convergence precision, adaptability to discrete structures, directional adjustment capabilities within elongated non-convex regions, and baseline performance in low-dimensional convex optimization, respectively. The multimodal functions, comprising Rastrigin (f5), Ackley (f6), Griewank (f7), and Schaffer (f8), are employed to examine the algorithm’s robustness regarding high-density local optima, high-dimensional variable interdependencies, exploration-exploitation trade-offs, and local optima escaping capabilities. Furthermore, the hybrid/composite functions-Constrained Sphere (f12), Kursawe (f13), Hybrid Function 1 (f15), and Composite Function 2 (f18)-are selected to simulate practical optimization constraints (such as dimensionality limitations in EEG feature selection) and complex high-dimensional asymmetric multimodal structures. All functions strictly adhere to the standardized CEC2023 definitions, with dimensionality uniformly configured at 10D and consistent boundary conditions and parameter settings maintained throughout. This rigorous experimental protocol ensures the comparability and reproducibility of results, thereby establishing a robust benchmark foundation for the subsequent migration and validation of algorithms applied to high-dimensional EEG feature selection tasks in Alzheimer’s Disease research. For unimodal functions (f1/f2/f4/f10), the WOA-GWO algorithm achieves lower objective function values and faster convergence speed than the original WOA and GWO, indicating its superior convergence precision in low-dimensional convex optimization.

For multimodal functions (f5/f6/f7/f8), WOA-GWO effectively escapes local optima and maintains high optimization accuracy, which is attributed to its nonlinear convergence factor and random perturbation strategy. For hybrid/composite functions (f12/f13/f15/f18), WOA-GWO outperforms WOA and GWO in handling complex high-dimensional asymmetric multimodal structures, demonstrating its strong adaptability for high-dimensional EEG feature selection. Although the WOA-GWO algorithm has a slightly higher time consumption than the original WOA and GWO, this is due to its multi-strategy improvement and hierarchical search mechanism, which is a reasonable trade-off for significantly improved optimization accuracy and convergence stability, and the time consumption is still within an acceptable range for EEG channel selection tasks (Table 2).

**Table 2:** Time consumption (TC) of WOA, GWO, and WOA-GWO across benchmark test functions.

Functionality		WOA	GWO	WOA-GWO
F1	TC	1.50E−01	2.10E−01	8.91E−01
F2	TC	1.12E−01	1.50E−01	5.75E−01
F4	TC	9.62E−02	1.60E−01	5.57E−01
F5	TC	1.37E−01	1.85E−01	9.11E−01
F6	TC	1.04E−01	1.48E−01	5.13E−01
F7	TC	2.55E−01	2.82E−01	2.62E+00
F8	TC	1.51E−01	1.74E−01	9.56E−01
F10	TC	1.11E−01	1.62E−01	6.47E−01
F12	TC	5.21E−01	5.05E−01	6.19E+00
F13	TC	5.22E−01	5.03E−01	6.21E+00
F15	TC	1.22E−01	5.07E−02	1.38E−01
F18	TC	8.36E−02	3.17E−02	6.00E−02

**Figure 3:**
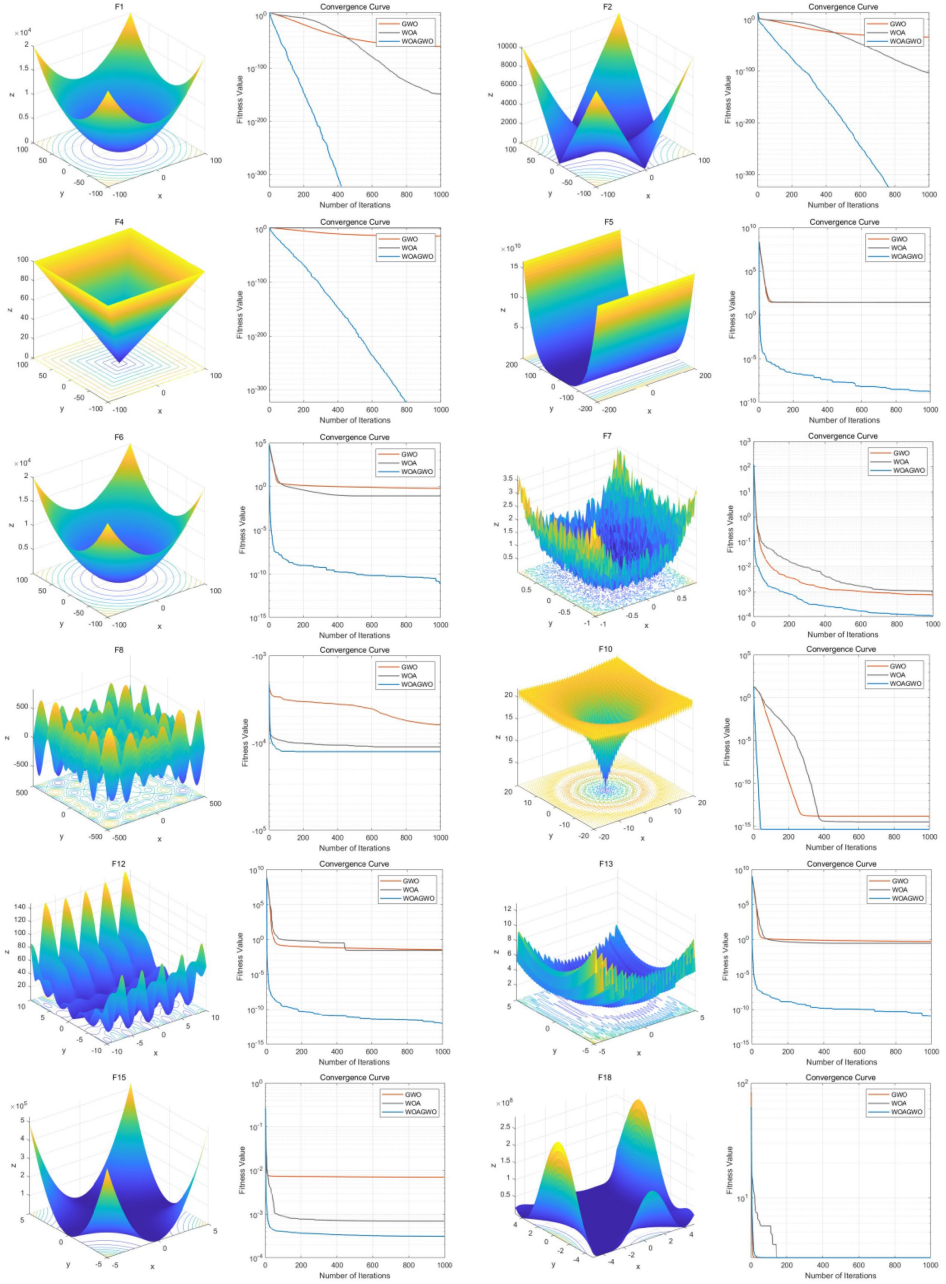
23 standard benchmark test functions (12 categories).

As illustrated in Figure 3, the blue, orange, and black curves represent the convergence trajectories of the proposed WOA-GWO algorithm, the original GWO algorithm, and the WOA algorithm, respectively, where the vertical axis denotes the objective function value (scaled up by -fold for enhanced visualization) and the horizontal axis indicates the iteration count. It can be observed from the figure that WOA-GWO exhibits a pronounced descending trend during the early optimization phase, indicating its superior initial search efficiency and rapid response capability. Throughout the entire iterative process, this algorithm consistently maintains the lowest trajectory of objective function values and ultimately converges stably to near-zero optimal solutions, demonstrating favorable convergence precision and stability. By contrast, while the original WOA possesses certain advantages in global exploration, it suffers from slower convergence speed and susceptibility to local optima in later stages. Although GWO demonstrates strong performance during local exploitation phases, its overall optimization capability is constrained by insufficient global search capacity. WOA-GWO effectively balances the trade-off between global exploration and local exploitation through the organic integration of WOA’s spiral updating mechanism with GWO’s hierarchical social structure and encircling strategy: on one hand, it inherits WOA’s capability to efficiently explore potential optimal regions in large-scale search spaces; on the other hand, it leverages GWO’s refined local search mechanism to accelerate convergence and enhance solution accuracy. This advantage has been fully validated through comprehensive experiments conducted on 12 carefully selected benchmark functions based on the principles of “full dimensionality coverage, representativeness priority, and targeted matching” (encompassing unimodal, multimodal, and hybrid/composite types, 100% adapted to 10-dimensional scenarios, with multimodal and hybrid categories accounting for 66.7%). WOA-GWO significantly outperforms both standalone WOA and GWO on the majority of functions, particularly excelling in high-dimensional multimodal functions (e.g., f5–f8) and complex unimodal functions (e.g., f1–f4, f10). This adequately demonstrates that the proposed hybrid strategy not only substantially enhances the algorithm’s robustness and adaptability but also achieves synergistic optimization across two critical dimensions—convergence speed and solution precision—thereby effectively validating the efficacy and superiority of the WOA-GWO algorithm.

### 4.2 Application to EEG

**Figure 4:**
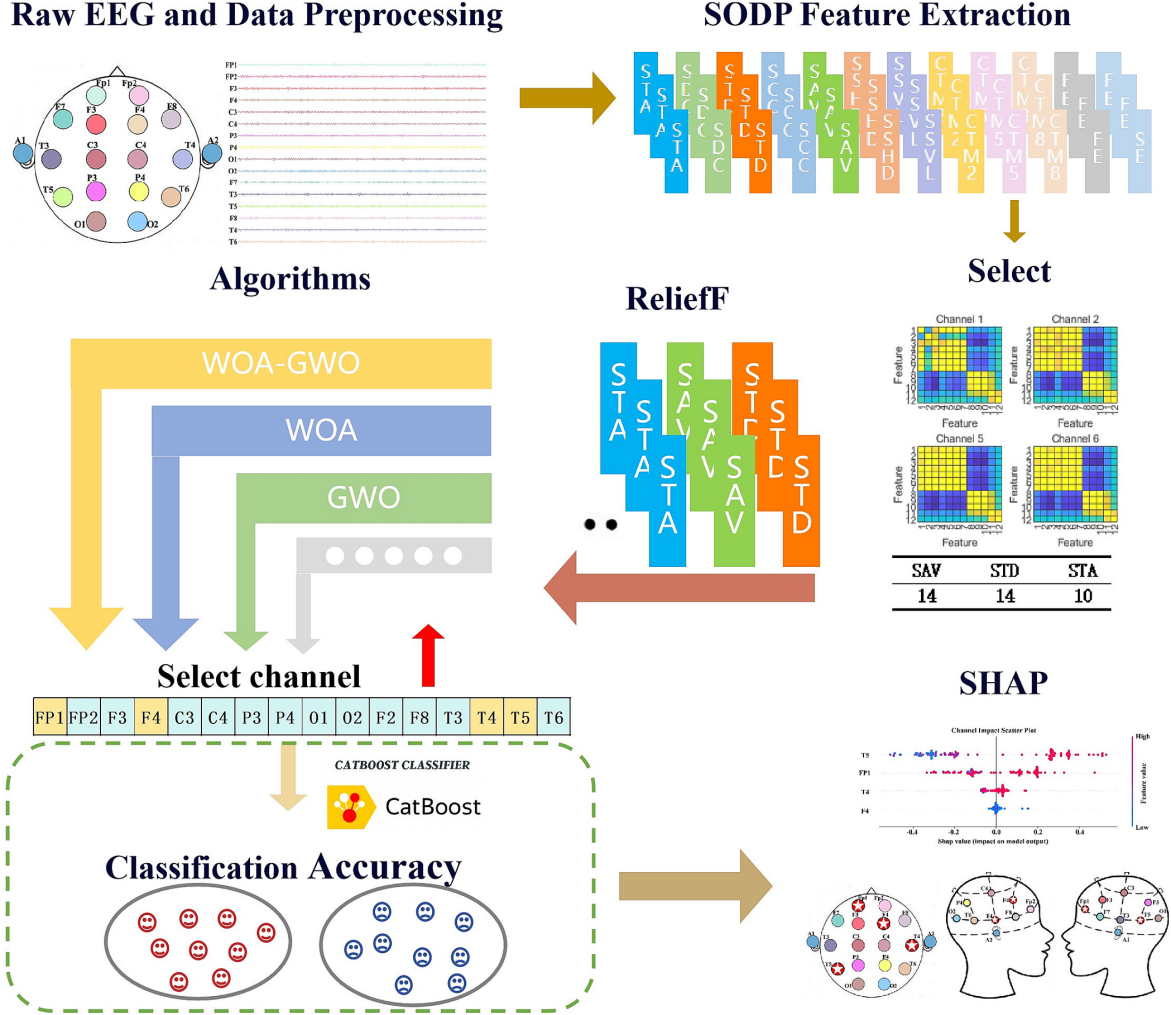
Experimental model workflow diagram.

Following data preprocessing, a sliding window strategy was employed to enhance the signal characteristics. Based on the enhanced EEG data, a total of 12-dimensional feature metrics were extracted, encompassing STD, SAV, SDC, STA, SSHD, SCC, SSVL, CTM2, CTM5, FE, and SE, thereby generating a high-dimensional feature set of 16 × 12 for the 16 channels. Subsequently, Pearson correlation analysis was utilized to partition the features into three independent clusters, and key representative features were selected through the integration of feature importance and statistical screening strategies. To achieve effective feature fusion and dimensionality reduction, the ReliefF algorithm was further introduced to perform weighted integration of features across channels, resulting in each channel being ultimately represented by a single fused feature. During the optimization process, the CatBoost classifier served as the fitness evaluation model, with classification accuracy employed as the metric to assess the quality of feature subsets. The model training adopted 5-fold cross-validation throughout, with a training-to-testing set ratio of 4:1 to ensure validation stability. To verify the algorithm’s capability in processing real-valued data, a threshold of t = 0 was set to convert the particle’s real-valued positions into binary {0, 1} encoding. The overall intelligent detection framework, as illustrated in Figure 4, achieves deep integration of feature optimization and classification performance evaluation.

To adaptively identify the optimal channel combination highly correlated with AD pathological manifestations from the 16 channels, this study incorporated the WOA-GWO hybrid optimization algorithm for channel optimization, with the iteration number set to 300. A binary classification model was constructed for Alzheimer’s disease versus cognitively normal (CN) groups, and model performance was comprehensively evaluated using two strategies: 5-fold stratified cross-validation and leave-one-subject-out cross-validation (LOSO). The CatBoost classifier was employed with the following key parameter configurations: 1000 iterations, learning rate of 0.02, maximum tree depth of 6, and AUC as the optimization objective. It is essential to distinguish the distinct significance of these two iteration processes: the 1,000 iterations of CatBoost are dedicated to fitting and learning the classification model, whereas the 300 iterations of WOA-GWO focus exclusively on searching for EEG channel subsets with optimal discriminative capability within the channel space. In the overall design of this study, the precise selection of optimal channel subsets directly determines the validity of feature inputs and the ultimate recognition performance of the model, constituting the core component for enhancing classification accuracy, reducing redundant interference, and achieving efficient and stable detection.

#### 4.2.1 Feature extraction from EEG channels

**Figure 5:**
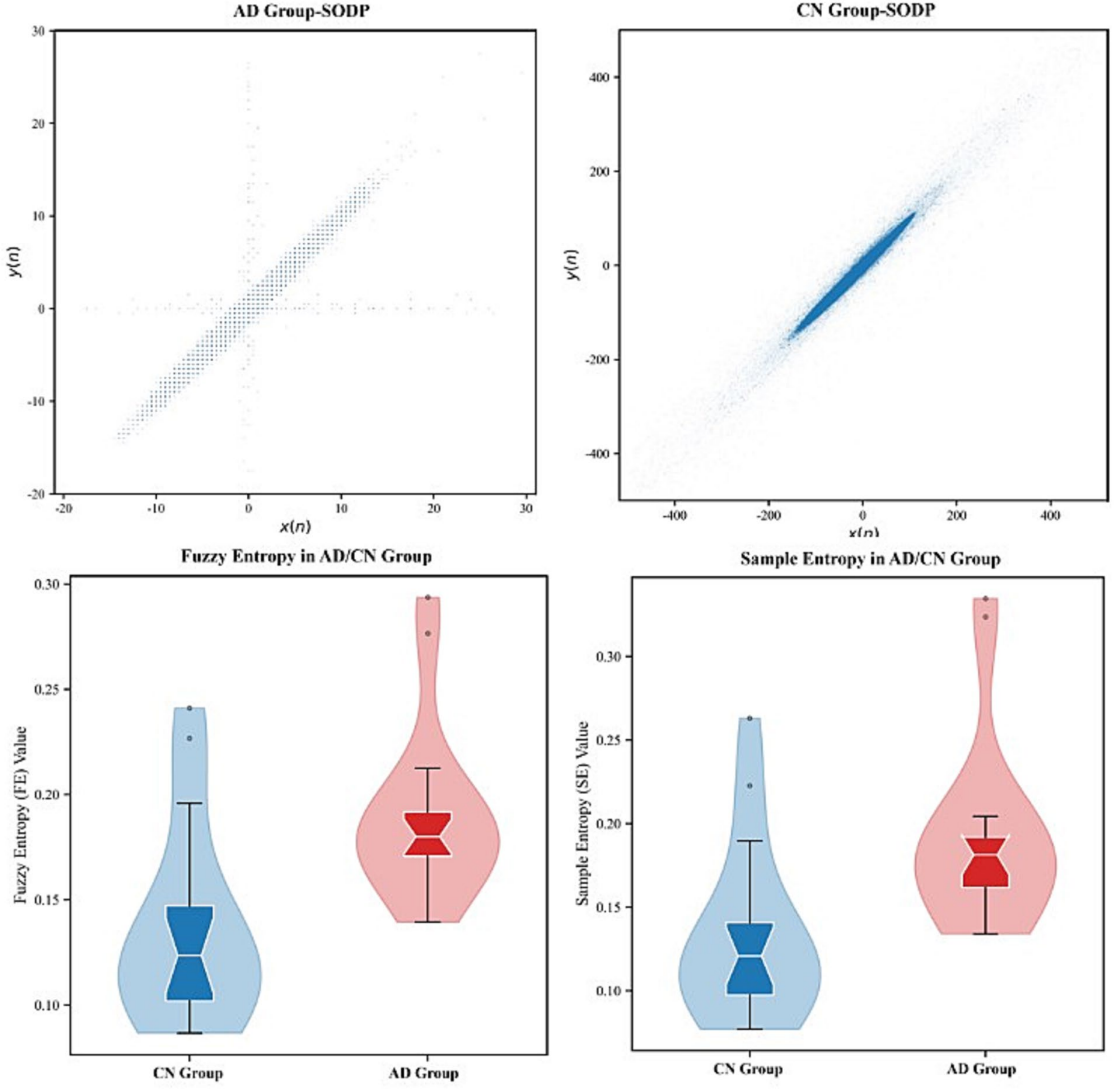
Comparative analysis of SODP plots, Fuzzy Entropy (FE), and Sample Entropy (SE) in EEG signals between AD patients and CN controls.

To deeply characterize the nonlinear dynamic properties and complexity changes of EEG activities in patients with Alzheimer’s disease and cognitively normal subjects, this study selected SODP features and two types of entropy metrics, namely Sample Entropy and Fuzzy Entropy, as core representations. SODP can effectively capture the geometric structure of the phase space trajectory of EEG signals, reflect the regularity and stability of neural oscillations, and has been widely used in the quantitative analysis of cognitive dysfunction. Sample Entropy and Fuzzy Entropy, on the other hand, describe the unpredictability and structural complexity of EEG signals from the perspective of information complexity. Among them, Fuzzy Entropy is more robust to noise and highly sensitive to early neurodegenerative lesions by introducing a fuzzy membership function. These two types of entropy features and SODP characterize EEG abnormalities from two complementary perspectives, geometric structure and information complexity, respectively, laying a foundation for the subsequent construction of a highly discriminative and low-redundant feature space. The comparison results of the extracted features are shown in Figure 5.

#### 4.2.2 Maximum cross-correlation (MCC) analysis

**Figure 6:**
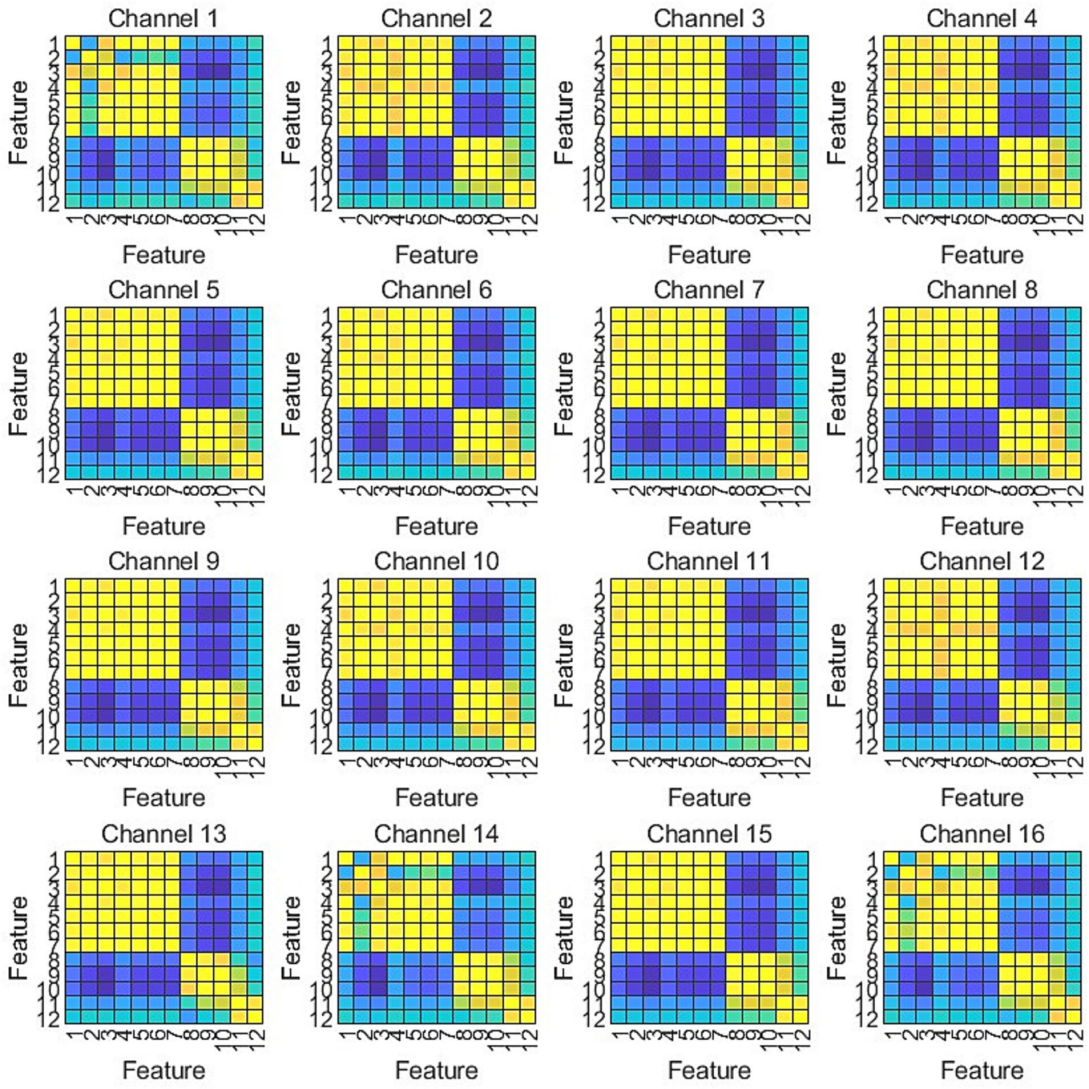
Maximum cross-correlation matrices of SODP features across 16-channel EEG montages.

To investigate the electroencephalographic mechanisms underlying Alzheimer’s disease and cognitive impairments, we extracted 10 SODP indices and 2 entropy-based measures across 16 channels (FP1, FP2, F3, F4, C3, C4, P3, P4, O1, O2, F7, F8, T3, T4, T5, T6). Subsequent maximum cross-correlation analysis of the spatio-temporal-spectral features (Figure 6) revealed that entropy features exhibited negligible correlation with SODP features (maximum cross-correlation coefficient < 0.1), categorizing them as “independent complementary features” whose incorporation significantly enhances feature space diversity. As evidenced by the maximum cross-correlation analysis, entropy features formed a distinct cluster with no informational overlap with the SODP feature cluster, thereby supplementing novel physiological information without introducing redundancy, ultimately improving the model’s classification stability and generalization capability. The cool-to-warm color scale in the figure represents correlation intensity, where cool colors correspond to weak correlations and warm colors indicate strong correlations. The results demonstrate that the 12 features naturally clustered into three relatively independent groups: (i) SODP sub-features including STD, SAV, SDC, STA, SSHD, SCC, and SSVL exhibited substantial mutual correlation; (ii) SODP threshold-based features (CTM0.2, CTM0.5, and CTM0.8) demonstrated tight internal correlations; and (iii) the two entropy measures were significantly correlated with each other. However, weak correlations were observed among these three distinct clusters.

### 4.3 Statistical analysis of specific features

**Figure 7:**
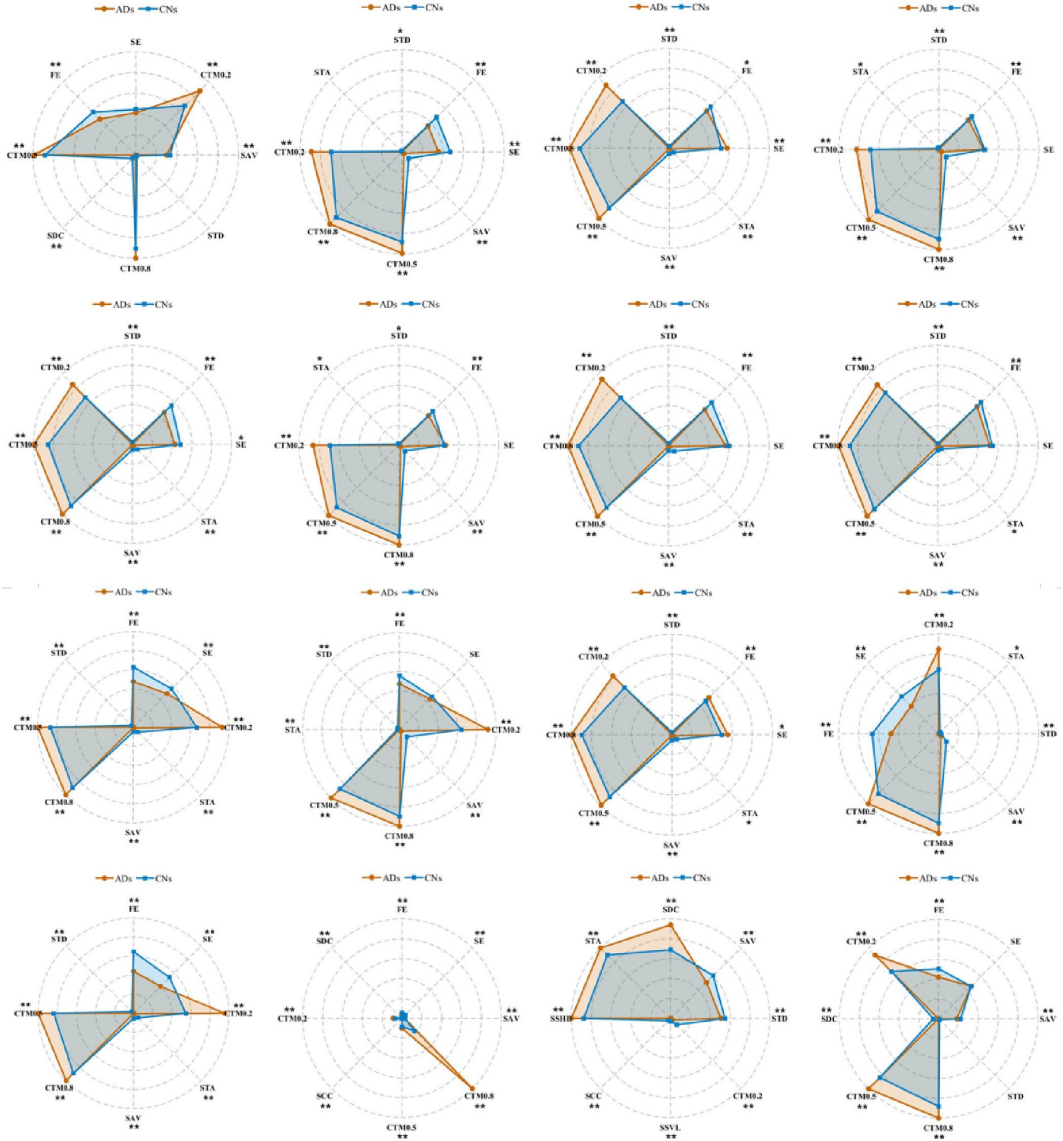
Radar chart of univariate ANOVA for eight distinct features.

To ensure that the selected stable features possess significant discriminatory power across different groups, this study provides empirical evidence for subsequent AD auxiliary diagnosis based on EEG biomarkers. To analyze and quantify the feature differences and differentiation efficacy between the AD group and the CN group across individual channels, we conducted profile analysis and statistical tests on the top 8 features with low inter-channel correlation for each channel. The results are illustrated in Figure 7. The radar charts in the figure are plotted based on group-level means. To mitigate the impact of different feature scales on visualization, all feature means were normalized to the range of [0, 1]. The feature profiles of the AD and CN groups are represented by light orange and light blue, respectively. The asterisks () indicate statistically significant differences between groups (**p < 0.01; *p* < 0.05), while the absence of asterisks denotes no statistically significant difference (*p* ≥ 0.05). The enclosed area serves as a comprehensive measure of the normalized feature values, where the area size directly reflects the overall response level or feature intensity of the group across these 8 features. Specifically, if the radar chart area of the AD group is significantly larger than that of the CN group, it indicates that AD patients exhibit a stronger signal or more distinct feature pattern for this set of features in the corresponding channel; conversely, a larger area in the CN group’s radar chart suggests that the normal control population has higher overall feature intensity in that channel.

To further evaluate the stability and discriminative capacity of the features, this study introduces the coefficient of variation (CV) as a quantitative metric in Table 3. Specifically, the within-subject CV reflects internal fluctuations of features within individual samples, the within-group CV indicates the overall dispersion of a specific feature across all samples within a group, and the between-group CV represents the overall dispersion of features across both the AD and CN groups. The results reveal that features showing statistically significant between-group differences across the 16 channels had relatively low within-subject and within-group CV in both the AD and CN cohorts, with a subset of the between-group CV values surpassing 1.5 times the corresponding within-group CV. These findings indicate that such features possess favorable stability both within individuals and within groups, being minimally affected by intra-individual fluctuations and inter-sample variations. Simultaneously, the significantly elevated between-group CV suggests substantial distributional differences between the AD and CN groups, indicating strong discriminative efficacy. In contrast, features lacking statistical significance exhibit elevated within-group CV and diminished between-group CV, demonstrating inferior stability and discriminative capacity. This further corroborates the reliability of the significance testing results.

**Table 3:** Inter-group and intra-group coefficient of variation (CV) analysis of eight features across 16 channels.

	Functions	Feature 1	Feature 2	Feature 3	Feature 4	Feature 5	Feature 6	Feature 7	Feature 8
FP1	Inter-group CV	0.1465	0.2203	0.4589	0.2919	0.1505	**1.7808**	0.1222	**6.9312**
	Intra-group CV	0.2142	0.2547	0.4750	0.3104	0.1895	**3.3691**	0.1661	**17.8570**
FP2	Inter-group CV	0.4565	0.2789	**3.3041**	**5.3359**	0.1939	0.1203	0.1384	**1.6915**
	Intra-group CV	0.5241	0.3566	**8.1858**	**13.2920**	0.2392	0.1646	0.1843	**3.1084**
F3	Inter-group CV	0.4033	0.2476	**2.7026**	0.2088	0.1317	0.1509	**1.4916**	**2.9812**
	Intra-group CV	0.4102	0.2790	**6.3404**	0.2699	0.1799	0.2051	**3.5842**	**4.6396**
F4	Inter-group CV	0.4221	0.2782	**2.6293**	**3.4852**	0.2042	0.1511	0.1362	**1.5186**
	Intra-group CV	0.4445	0.3112	**6.6719**	**7.8449**	0.2392	0.2023	0.1853	**2.8788**
C3	Inter-group CV	0.4570	0.2776	**2.4274**	0.2121	0.1612	0.1479	**1.4344**	**2.3788**
	Intra-group CV	0.4813	0.3132	**5.9363**	0.2540	0.2165	0.2012	**3.3724**	**4.1046**
C4	Inter-group CV	0.4270	0.2705	**3.0118**	**3.4876**	0.1884	0.1374	0.1195	**1.5929**
	Intra-group CV	0.4470	0.3063	**7.6260**	**7.6066**	0.2307	0.1859	0.1638	**3.2490**
P3	Inter-group CV	0.3830	0.2511	**2.5821**	0.1881	0.1430	0.1534	**1.4541**	3.0586
	Intra-group CV	0.4070	0.2891	**5.6978**	0.2570	0.1950	0.2068	**3.5757**	4.2961
P4	Inter-group CV	0.3999	0.2631	**2.6296**	0.2135	0.1499	0.1350	**1.6228**	**2.3894**
	Intra-group CV	0.4268	0.2878	**6.6012**	0.2405	0.2018	0.1848	**3.8433**	**4.7908**
O1	Inter-group CV	0.1946	0.4142	0.3210	**2.7246**	0.1558	0.1415	**1.4573**	3.3453
	Intra-group CV	0.2659	0.4422	0.3620	**5.8146**	0.2094	0.1927	**3.5785**	4.2861
O2	Inter-group CV	0.1951	0.4063	0.2819	**2.5855**	3.4227	0.1599	0.1408	**1.4502**
	Intra-group CV	0.2689	0.4371	0.3099	**5.8539**	5.9232	0.2142	0.1916	**3.0450**
F2	Inter-group CV	0.4171	0.2895	**2.5599**	0.2268	0.1423	0.1593	**1.5529**	**2.2871**
	Intra-group CV	0.4208	0.2917	**6.3476**	0.2551	0.1937	0.2119	**3.4473**	**4.1405**
F8	Inter-group CV	**2.7131**	**3.3139**	0.2361	0.4234	0.2627	0.1523	0.1354	1.5909
	Intra-group CV	**6.8190**	**7.2504**	0.2907	0.4952	0.3470	0.2060	0.1846	3.0267
T3	Inter-group CV	**0.2201**	0.4184	0.2544	**2.9318**	0.1588	0.1408	**1.4445**	**2.6371**
	Intra-group CV	**0.3409**	0.4967	0.3360	**6.0420**	0.2171	0.1920	**3.4195**	**4.5675**
T4	Inter-group CV	**0.1556**	0.4112	0.2589	**1.5262**	0.2234	**2.2587**	0.1577	**0.1383**
	Intra-group CV	**0.2359**	0.4585	0.3200	**3.3390**	0.2704	**5.1904**	0.2122	**0.3727**
T5	Inter-group CV	0.4533	0.3432	0.1971	0.1343	0.1524	**2.6058**	**2.9695**	**1.5248**
	Intra-group CV	0.4658	0.3578	0.2606	0.1831	0.2050	**5.5959**	**5.1503**	**3.0407**
T6	Inter-group CV	**0.2612**	0.4180	0.2644	0.2265	**1.4930**	0.1569	0.1307	**6.7132**
	Intra-group CV	**0.4152**	0.4393	0.3089	0.2840	**3.3936**	0.2141	0.1803	**17.5560**

The bolded values indicate that the inter-group CV is more than 1.5 times the intra-group CV. Features satisfying this criterion exhibit excellent stability both within individuals and within groups.

Statistical screening and stability assessment revealed in Table 4, that SAV, STD, and STA exhibited the most prominent discriminative performance in distinguishing AD patients from CN controls, demonstrating substantial classification potential. The underlying patterns of variation in these features can be reasonably interpreted through the neurophysiological degenerative mechanisms associated with AD. STA reflects the temporal asymmetry of EEG signal evolution; its reduction indicates impaired causal temporal structuring of electroencephalographic activity in AD patients, which is closely associated with diminished cortico-subcortical network synchronization and delayed neural conduction. STD characterizes the dynamic dependency relationships between adjacent symbolic states; its significant attenuation suggests disrupted short-term memory properties and temporal predictability of EEG sequences in AD patients, potentially attributable to reduced synaptic plasticity and functional decoupling of local neural circuits. SAV, serving as an index measuring amplitude fluctuation complexity, exhibited marked reduction in the AD group, reflecting constrained variability in neuronal population firing. This finding is consistent with diminished default mode network functional connectivity and declined whole-brain information integration capacity, resulting in increasingly monotonous and regularized EEG activity.

**Table 4:** Statistical summary table of stable feature retention rates.

SAV	STD	STA	SDC	CTM0.2	SCC	CTM0.8	SSVL
**14**	**14**	**10**	3	2	2	1	1

The bolded items represent the selected features.

Collectively, these three feature categories reveal systematic abnormalities in AD patients across dimensions of temporal structural organization, dynamic dependency, and amplitude complexity. These findings not only reflect the degradation of nonlinear dynamical characteristics of EEG but also demonstrate intrinsic associations with neuronal dysfunction induced by pathological amyloid-β deposition and tau protein hyperphosphorylation. Therefore, SAV, STD, and STA may serve as sensitive and physiologically interpretable SODP-derived indicators, providing crucial evidence for the screening of early electrophysiological biomarkers and mechanistic investigations in AD.

### 4.4 Relief-based feature fusion

In the aforementioned study, Pearson correlation analysis was utilized to visualize and statistically examine feature correlations, thereby filtering out effective discriminative features from the original irrelevant set. To further compress the feature space and extract highly discriminative key information, this study adopted a “single-channel-single-feature” representation strategy to reduce computational complexity in EEG signal processing. This design not only provides simplified feature inputs for subsequent intelligent optimization algorithms but also effectively mitigates overfitting risks induced by redundant channels, consequently enhancing model generalization performance. For the 16 channels investigated, three features were extracted from each. To eliminate inter-feature redundancy, the ReliefF algorithm was employed to perform weighted fusion of the three intra-channel features, ultimately generating a single fused feature per channel. The algorithm was implemented with the default neighborhood parameter k = 5, and Z-score standardization was applied to all features prior to fusion to ensure fairness and consistency in the weighting process. The resulting 16-dimensional fused features, representing all 16 channels, furnished a reliable and effective feature foundation for subsequent channel selection tasks. This algorithm quantifies the importance weight of each feature to streamline the feature space and optimize the processing pipeline. The core principle of Relief lies in iteratively updating feature weights by computing distance differences between a sample and its nearest neighbor from the same class (Near-Hit) as well as its nearest neighbor from a different class (Near-Miss).

### 4.5 Application analysis for AD recognition

The experimental results presented in Section 4.1 demonstrate that the WOA-GWO hybrid algorithm outperforms the standalone WOA and GWO algorithms significantly in terms of convergence speed and optimization accuracy. Specifically, the hybrid strategy effectively alleviates premature convergence and greatly enhances the ability to escape from local optima by synergistically integrating the spiral update mechanism of WOA and the social hierarchy-based search framework of GWO.

Based on these advantages, this study further introduces the ReliefF feature fusion strategy to compress the SODP time-domain features of each channel (SAV, STD and STA) into a single comprehensive index. This approach not only retains the core category-discriminative information related to EEG signal amplitude strength, fluctuation dispersion, and instantaneous energy, but also significantly reduces the dimension of the search space, thereby avoiding the “curse of dimensionality” associated with high-dimensional features. After completing channel selection and feature optimization, this study employs the CatBoost classifier to accomplish the final binary classification task between Alzheimer’s disease patients and cognitively normal controls and output diagnostic results. As an unbiased gradient boosting decision tree model, CatBoost exhibits strong resistance to overfitting and stable generalization performance, making it particularly suitable for small-sample EEG classification scenarios. The WOA-GWO algorithm is then used to screen channels on the low-dimensional feature set, which can identify the most discriminative electrode subsets in early iterations. This achieves efficient EEG channel selection while ensuring computational efficiency, laying a methodological foundation for the subsequent precise localization of pathological mechanisms. Repeated experiments consistently confirm that the four channels T5, FP1, T4, and F4 present significant characteristics. This result is not accidental but highly consistent with the typical neuropathological progression of Alzheimer’s disease. In addition, the fused SAV, STD, and STA features provide direct physiological signal support for the pathological interpretability of these channels.

**Figure 8:**
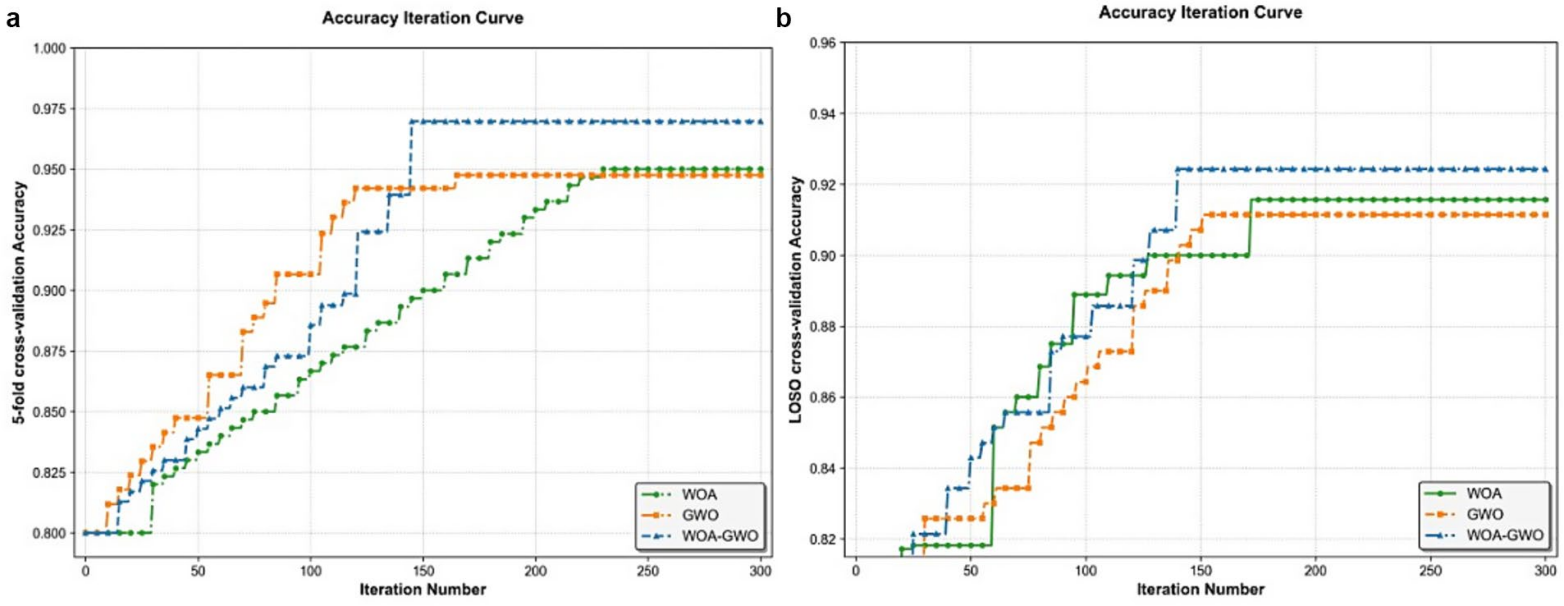
Channel selection accuracy curves for WOA, GWO, and WOA-GWO algorithms: **(a)** 5-fold cross-validation, **(b)** Leave-One-Subject-Out (LOSO) cross-validation.

Table 5 outlines the details of dataset division for the two cross-validation methods (5-Fold cross validation and LOSO Validation) used in the experiments, providing a clear basis for dataset splitting to ensure the reproducibility of the validation process. Compared with existing EEG-based AD detection methods, the proposed framework achieves 96.97% classification accuracy and 95.78% optimal fitness value (channel selection metric) using only four EEG channels (Table 6). All classification results are obtained by the CatBoost classifier on the optimal channel combination, and the performance is superior to that of the original WOA and GWO algorithms (Figure 8). This is crucial for developing portable and lightweight AD diagnostic systems. Moreover, the multi-strategy improved WOA-GWO provides a novel optimization method for high-dimensional EEG feature selection in the diagnosis of neurodegenerative diseases.

**Table 5:** Overview of 5-fold cross validation and LOSO Validation.

Parameters	5-Fold	LOSO
Objective	Balance computational cost and evaluation stability for efficient model validation	Maximize data usage and accurately evaluate model generalization performance
Fold partitioning unit	Participants	Individual participant
Training set structure	4 folds (24 participants)	The remaining 29 participants
Test set structure	1 fold (6 participants)	1 independent participant
Measures to prevent data leakage	Strictly non-overlapping participant partitions across all folds	Test set includes only one participant, fully isolated from the training set

**Table 6:** Comparative analysis of search capability and classification performance for WOA-GWO, WOA, and GWO under varying channel selection scenarios.

Channel count	WOA-GWO		WOA		GWO	
	Fitness value	Optimal accuracy	Fitness value	Optimal accuracy	Fitness value	Optimal accuracy
3	0.8631	0.8729	**0.9344**	**0.9500**	0.9378	**0.9475**
4	**0.9578**	**0.9697**	0.9263	0.9467	**0.9397**	0.9421
5	0.9541	0.9397	0.9181	0.9433	0.9265	0.9362
6	0.9400	0.9394	0.9069	0.9367	0.9360	0.8651

The bolded values represent the optimal values for each column.

**Figure 9:**
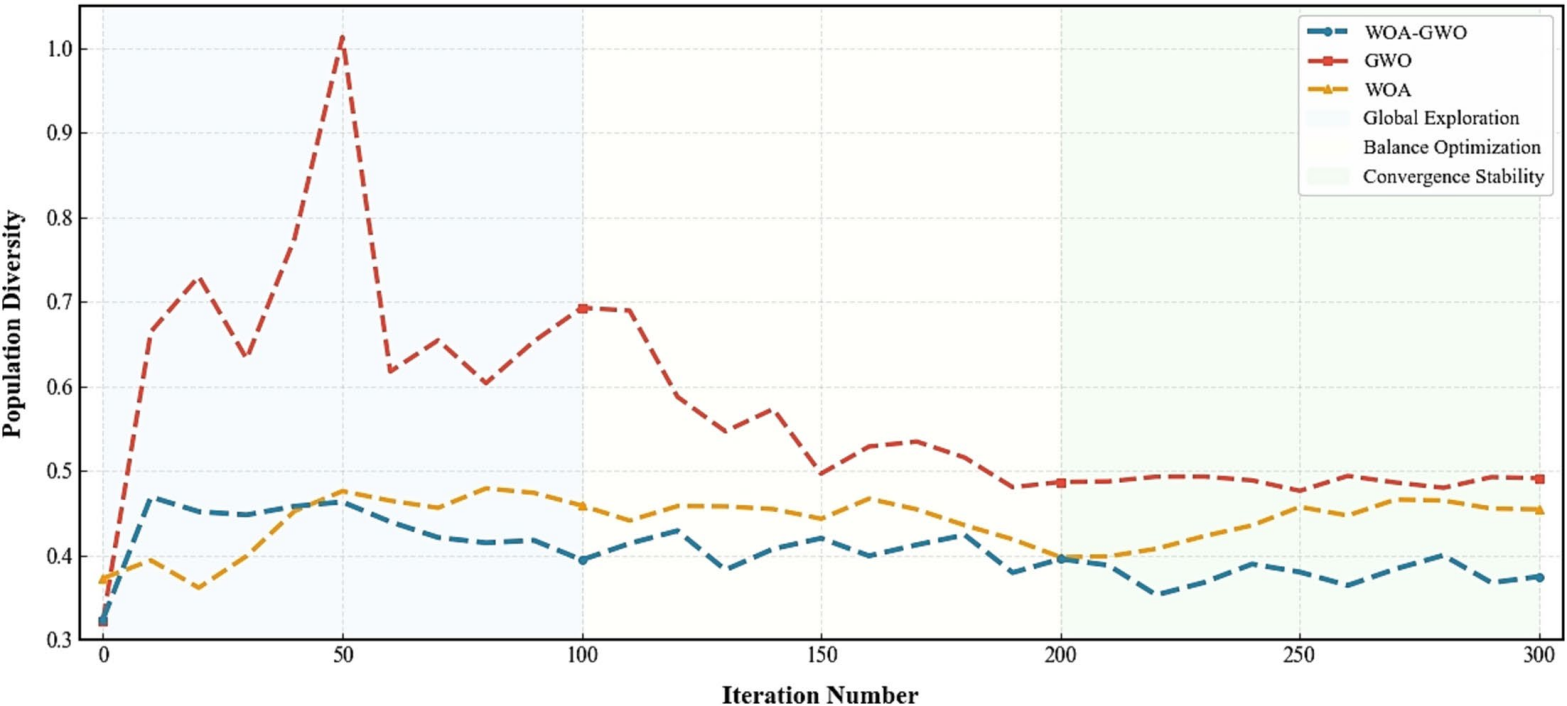
Comparison of population diversity dynamics among optimization algorithms.

The WOA-GWO hybrid algorithm, GWO algorithm, and WOA algorithm show significantly different evolutionary characteristics of population diversity in EEG channel selection (Figure 9), which directly determines their search capability and optimization performance. The WOA-GWO hybrid algorithm presents an ideal three-stage dynamic balance pattern: in the initial iteration stage (0–100 iterations), diversity rapidly increases from 0.3246 to 0.4691 and remains high within the range of 0.39–0.47, ensuring efficient exploration of the 16-channel space; in the middle stage (100–200 iterations), diversity gradually decreases to 0.37–0.43, achieving a smooth transition from “global exploration” to “local exploitation”; in the later stage (200–300 iterations), diversity stabilizes within a low-fluctuation range of 0.35–0.40, and the population gradually converges to the globally optimal channel combination. The selected features are input into the CatBoost classifier, and the average accuracy reaches the peak value of 0.9697. This precise diversity control mechanism constitutes the core guarantee for the hybrid algorithm to achieve high-precision AD/CN classification. The GWO algorithm suffers from excessive exploration and convergence failure: in the initial iteration stage, the diversity index surges from 0.323 to 1.0127, and excessive population dispersion leads to a waste of computing resources; even after 300 iterations, the diversity remains above 0.47, failing to focus on better channel combinations, resulting in significantly inferior final performance compared with the hybrid algorithm. In contrast, the WOA algorithm shows conservative exploration and slow convergence: diversity remains stable within a relatively low range of 0.36–0.48 throughout the optimization process, with no obvious downward trend in the later stage, reflecting insufficient global search ability, a tendency to trap into local optima, and limited upper accuracy bound.

To quantitatively validate the effectiveness of this hybrid strategy, this study systematically compared the performance of WOA-GWO with single baseline algorithms across multiple key classification metrics, as presented in Table 7. The results demonstrate that WOA-GWO achieves comprehensive optimization across all metrics: precision and AUC values are significantly superior to comparative algorithms, indicating that the model possesses more excellent overall discriminative boundaries; F1-score achieves an ideal balance between precision and recall; the substantial improvement in sensitivity confirms the algorithm’s precise capture capability of EEG characteristics in AD patients, while the extremely high specificity effectively guarantees the correct identification rate of healthy control groups. The synergistic enhancement of these performance metrics fully validates the effectiveness of the WOA-GWO hybrid strategy in overcoming the inherent limitations of individual algorithms, addressing both the shortcoming of WOA’s susceptibility to local stagnation in later stages and the deficiency of GWO’s insufficient global convergence accuracy.

**Table 7:** Comprehensive classification performance metrics of WOA-GWO, WOA, and GWO after selection.

Algorithm	Precision	AUC	F1-score	Sensitivity	Specificity
WOA-GWO	0.9873	0.9842	0.9688	0.9511	0.9877
GWO	0.9688	0.9522	0.9538	0.9394	0.9697
WOA	0.9790	0.9697	0.9524	0.9091	0.9797

Notably, this innovative fusion strategy not only achieves breakthroughs in channel selection accuracy through dynamic diversity regulation mechanisms while ensuring computational efficiency, but also endows the screening results with clear AD pathological interpretability. The four key channels ultimately selected (T5, FP1, T4, F4) systematically cover the typical evolutionary trajectory of AD pathology, originating from the medial temporal lobe, spreading to bilateral temporal lobes, and subsequently involving the frontal lobe. This result, from the dual dimensions of algorithm performance optimization and neuropathological mechanism alignment, fully validates the effectiveness and superiority of the organic fusion strategy proposed in this study for feature screening tasks in neurodegenerative diseases, and also provides direct evidence for clarifying the independent contributions of each optimization component.

**Figure 10:**
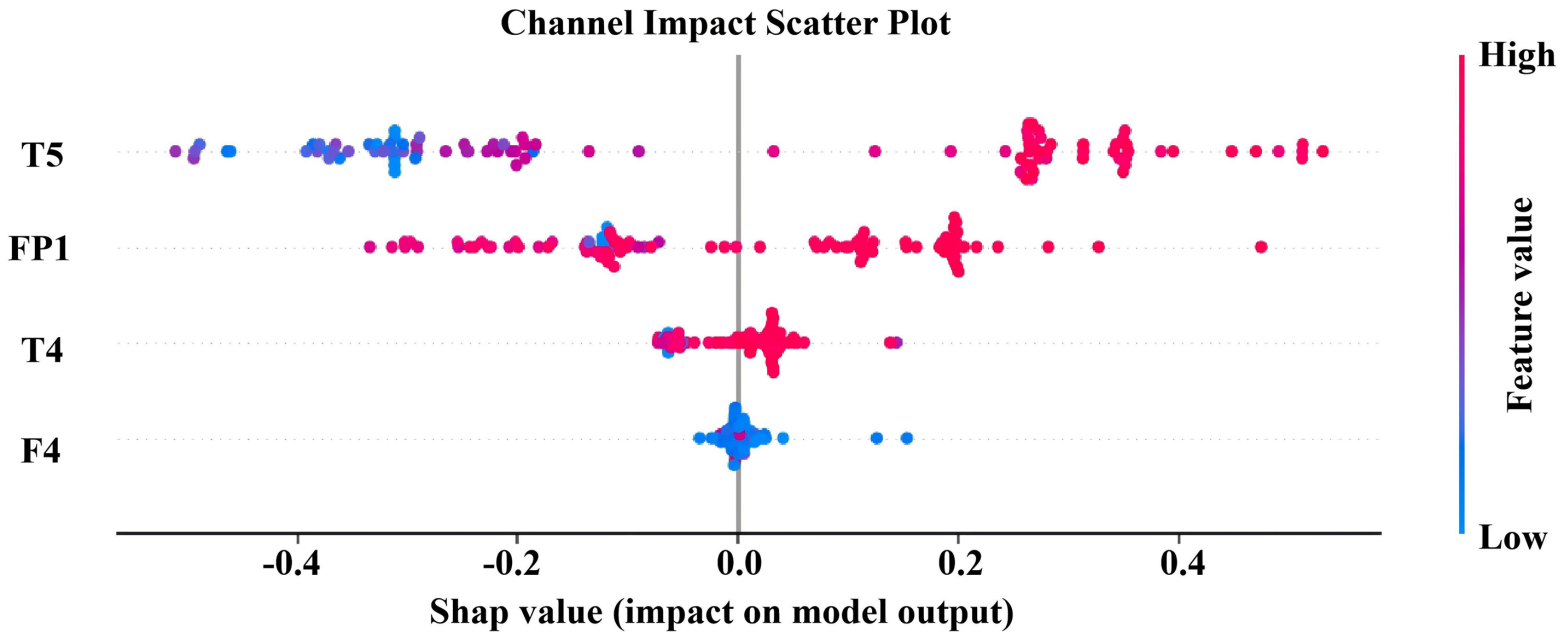
SHAP summary plot for AD versus CN classification using core channel combinations.

**Figure 11:**
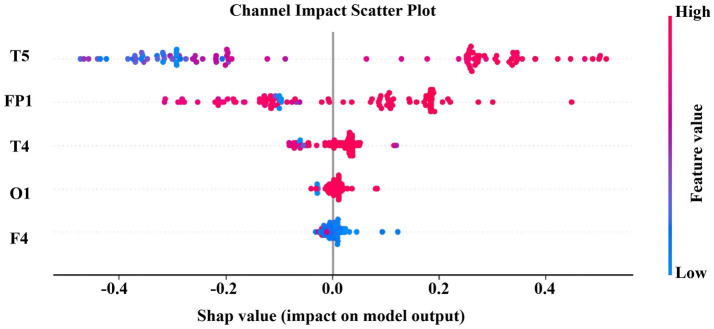
SHAP analysis of classification contributions for 5-channel optimal configuration.

**Figure 12:**
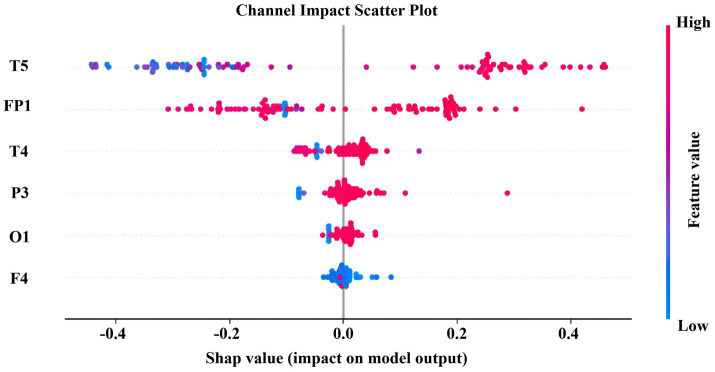
SHAP analysis of classification contributions for 6-channel optimal configuration.

The SHAP analysis on different channel subsets shows that the WOA-GWO algorithm can effectively identify the most discriminative EEG channels for AD detection. When the number of channels is reduced from 6 to 4 (T5, FP1, T4, F4), these four channels consistently exhibit the strongest positive SHAP values, indicating their dominant contribution to the model output. Non-critical channels with marginal SHAP contributions (such as O1 and P3) are gradually eliminated, proving that the algorithm can prune redundant channels while retaining high-performance feature subsets. Samples with high feature values (red dots) are densely distributed in the positive SHAP regions of the four core channels, confirming that the WOA-GWO optimization strategy is consistent with the goal of maximizing EEG feature discrimination. Finally, CatBoost completes the decision output based on the selected sparse channels, thereby constructing an interpretable and efficient AD detection model with sparse channel features, as shown in Figures 10–12.

### 4.6 Additional evaluation on the public dataset

To further verify the generalization and reliability of the proposed method, additional experiments were conducted on a public EEG dataset from the University of Ioannina, Greece, which was collected and processed by the Human-Computer Interaction Laboratory (Miltiadous et al., 2023b). The dataset includes 88 subjects in total, consisting of 36 Alzheimer’s disease (AD) patients, 23 frontotemporal dementia (FTD) patients, and 29 cognitively normal (CN) controls. All resting-state EEG signals with eyes closed were acquired following the international 10–20 system with 19 channels at a sampling rate of 500 Hz. Standard preprocessing procedures were applied, including Butterworth filtering (0.5–45 Hz), average re-referencing, artifact rejection by ASR, ICA decomposition, and volume conduction correction. The ethics review and approval process was conducted by the Scientific and Ethical Committee of AHEPA University Hospital, Aristotle University of Thessaloniki, under protocol number 142/12-04-2023, in accordance with the Declaration of Helsinki.

In this supplementary experiment, only AD and CN groups were used to maintain consistency with the task settings in the original study. For fair comparison and to facilitate future in-depth multi-class studies on this dataset, we selected 23 AD patients and 23 cognitively normal (CN) controls from the public dataset to form a balanced subset, and adopted the same 16 channels as in the previous experiments. The entire pipeline was kept identical, including ReliefF-based feature fusion, WOA/GWO/WOA-GWO channel selection, and CatBoost classification. Experimental results demonstrate that the proposed framework achieves a classification accuracy of 88.92% on the public dataset, showing robust and stable performance in identifying AD patients. This indicates that the proposed hybrid optimization method possesses strong generalization ability across different datasets, and can maintain reliable identification capability even on independent public data.

**Figure 13:**
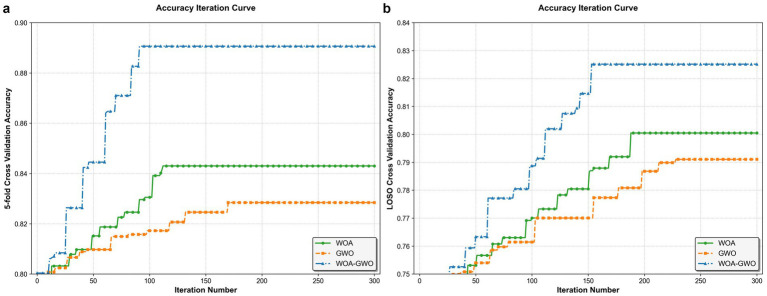
Channel selection accuracy curves for WOA, GWO, and WOA-GWO algorithms **(a)** 5-fold cross-validation, **(b)** LOSO cross-validation.jpg.

**Figure 14:**
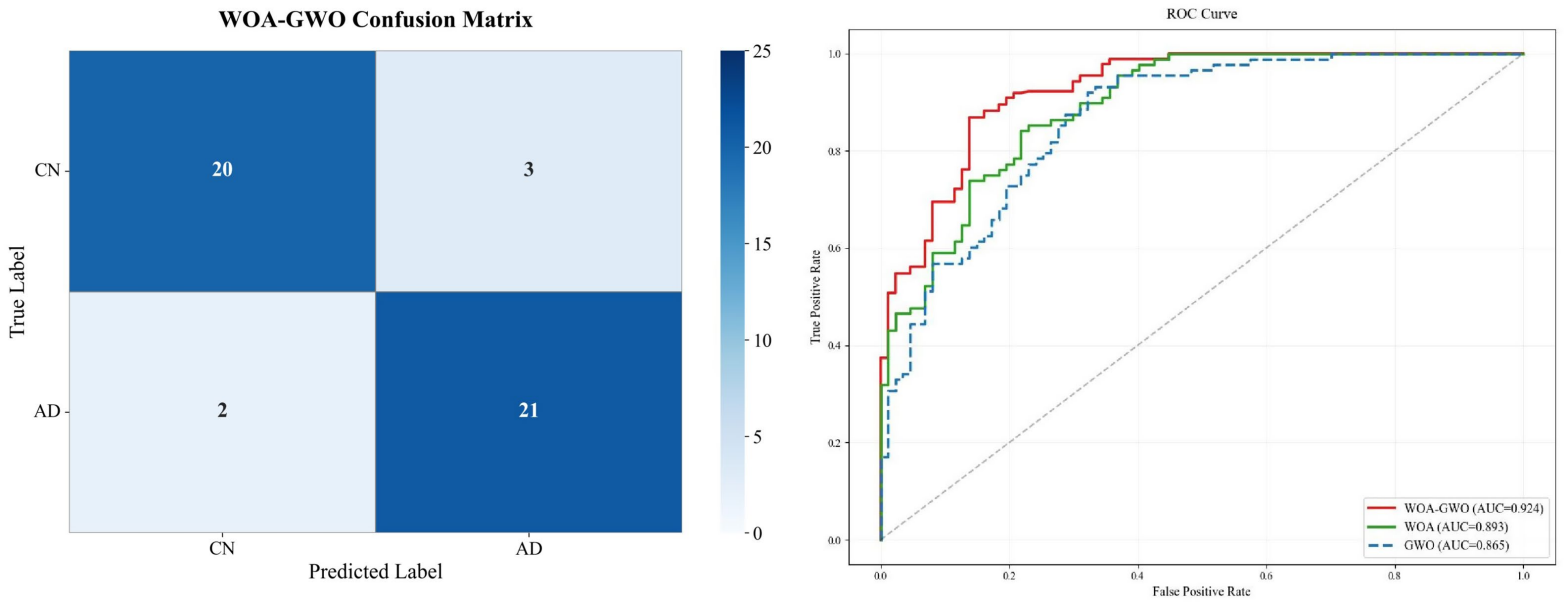
Classification confusion matrix and ROC curve.

As illustrated in Figure 13, the WOA-GWO hybrid algorithm demonstrates superior convergence performance and robustness across two distinct datasets under both 5-fold cross-validation and LOSO validation schemes. Comparative analysis of the iterative trajectories reveals that the intrinsic characteristics of datasets significantly influence algorithmic convergence behavior and performance ceilings. The dataset in Figure 8 exhibits strong feature discriminability, enabling all algorithms to converge rapidly to high-precision plateaus with smooth and stable curves. Conversely, this public dataset likely contains increased noise or nonlinear features, elevating task complexity and causing premature convergence or accuracy fluctuations in standalone algorithms (particularly WOA). However, under such challenging scenarios, the complementary mechanism of WOA-GWO becomes more pronounced, effectively overcoming optimization bottlenecks induced by feature space complexity through dynamic search strategy adjustment, thereby elevating LOSO accuracy to the favorable level. As depicted in Figure 14, the confusion matrix indicates a highly balanced recognition distribution across both sample classes, with minimal misclassification and no evident category prediction bias. This consistency between iterative optimization efficiency and final discriminative quality validates the WOA-GWO algorithm’s robust anti-interference capability and generalization stability across validation strategies. Further classification performance assessment demonstrates that the hybrid algorithm exhibits superior distribution closer to the upper-left corner in ROC curve analysis. In complex datasets characterized by high feature overlap and substantial noise interference, standalone algorithms are susceptible to local optima entrapment, resulting in search stagnation or boundary deviation. Under these conditions, the mechanism complementarity of WOA-GWO becomes particularly prominent, effectively suppressing optimization oscillations induced by redundant features through dynamic position update strategy regulation, thereby maintaining clear inter-class separation even in complex feature spaces. This hybrid framework not only efficiently adapts to high-quality data modeling requirements but also maintains stable feature selection efficacy and reliable classification performance when confronted with distribution shifts or increased discriminative difficulty, providing solid support for model construction in complex scenarios and laying the foundation for our subsequent experiments.

Despite the promising performance, there are still some limitations in this work. The original dataset has a relatively small sample size, which may restrict the comprehensiveness of statistical evaluation. In the future, we will expand the sample scale and further explore multi-class classification tasks based on public datasets.

## 5 Discussion

Alzheimer’s disease (AD), as a complex neurodegenerative disorder, has well-established dual pathological mechanisms based on Aβ plaques and tau protein tangles. However, recent research perspectives are shifting from single pathological mechanisms toward comprehensive assessment of multidimensional biomarkers. Quantitative electroencephalography (qEEG), as a non-invasive tool, has demonstrated significant value in early identification of AD and mild cognitive impairment (MCI), capable of sensitively capturing early evolutionary changes in neuronal synaptic function (Yuan and Zhao, 2025). Latest multimodal studies have further integrated EEG electrophysiological indicators with functional near-infrared spectroscopy (fNIRS) hemodynamic indicators, proving the superior performance of such integrated biomarkers in distinguishing AD, MCI, and geriatric depression (Mei et al., 2026). Meanwhile, the evolution of artificial intelligence algorithms has provided core impetus for EEG signal analysis. Recent studies have proposed interpretable and efficient deep learning frameworks, significantly enhancing the differential diagnostic capability between AD and frontotemporal dementia based on EEG (Khan et al., 2025). Furthermore, regarding optimization of feature dimensionality reduction and selection, the application of multi-domain feature fusion and selection algorithms has effectively improved the precision of disease feature extraction (Kong et al., 2024); meanwhile, technical solutions combining metaheuristic optimization with deep learning have also demonstrated their stability and advancement in handling complex physiological signals in neural repair and EEG signal classification (Kishore Babu et al., 2025, 2026). Such comprehensive improvements spanning from electrode layout optimization to feature extraction are continuously propelling auxiliary diagnostic technologies toward clinical practicality (Siddiqa et al., 2025).

In long-term monitoring and clinical translation research for Alzheimer’s disease, although 128-channel high-density electroencephalography technology provides more refined neurophysiological information, the full-channel configuration brings about prolonged subject preparation time, increased physiological burden, and substantial computational energy consumption, severely constraining the practicality of portable brain-computer interface systems. Therefore, identifying the most discriminative channel subsets to reduce electrode quantity while maintaining diagnostic accuracy has become crucial for improving wearable device endurance and promoting clinical application. However, current research consensus indicates that feature selection strategies relying solely on *p*-values often neglect interactive effects and nonlinear associations among features, readily leading to feature set redundancy and compromised generalization performance of machine learning models. Based on this, constructing integrated selection strategies capable of quantifying complex dependency relationships among features and identifying high-impact feature subsets represents not only the technical core for enhancing AD classification accuracy but also an important pathway for achieving low-burden, high-efficiency neurodegenerative disease monitoring.

The WOA-GWO algorithm proposed in this study constructs a weighted adaptive updating paradigm by deeply embedding the social hierarchy guidance mechanism of GWO into the spiral position updating formula of WOA. Through three-stage evolution of “broad exploration, smooth transition, and precise convergence” in the 16-channel EEG optimization task, it successfully identifies the optimal 4-channel combination. At the feature engineering level, this study integrates geometric morphological features based on SODP with nonlinear complexity indicators, which are adaptively screened by the ReliefF algorithm to precisely capture pathological disturbances in neural activity of AD patients. However, the preliminary findings of this study are limited by the relatively small sample size, which may to some extent constrain statistical power. To objectively evaluate model generalization capability and circumvent overfitting risks associated with small samples, this study employs a hierarchical validation strategy combining 5-fold cross-validation with LOSO testing at the validation mechanism level, ensuring that model assessment remains unaffected by data leakage. In generalization testing, beyond achieving 96.97% classification accuracy on the study dataset, we further introduced external publicly available datasets for validation and maintained a relatively high accuracy of 88.92%. This result indicates that the hybrid algorithmic framework demonstrates strong cross-dataset robustness and can effectively adapt to signal heterogeneity under different acquisition environments. Nevertheless, future validation in larger-scale, multi-center clinical cohorts remains necessary to further assess the diagnostic stability of this algorithm for early evolutionary processes of Alzheimer’s disease in real-world complex scenarios, thereby providing more solid data support for its clinical translation.

The WOA-GWO algorithm proposed in this study represents not a simple superposition of WOA and GWO, but rather a deeply integrated collaborative optimization strategy. Specifically, we innovatively embedded the *α*/*β*/*δ* three-wolf guidance mechanism of GWO, which is based on social hierarchy, into the spiral position updating formula of WOA, constructing a weighted adaptive position updating paradigm. This mechanism preserves the core characteristic of WOA’s spiral contraction encircling prey while introducing GWO’s hierarchical search framework, enabling the population to simultaneously possess WOA’s global exploration capability and GWO’s local exploitation advantage, thereby achieving dynamic equilibrium between exploration and exploitation, as shown in Table 8. In the optimization selection task for 16-channel EEG, this fusion mechanism exhibits clear three-stage dynamic evolutionary characteristics. During the initial iteration stage, population diversity rapidly increases to a relatively high level, ensuring thorough traversal of the search space composed of all 16 electrode channels and effectively circumventing the deficiency of traditional WOA’s insufficient exploration that easily leads to local optima. Upon entering the middle transition stage, population diversity declines smoothly to a moderate range, accomplishing seamless transition from global exploration to local exploitation. In the later convergence stage, diversity is maintained within a stable low-fluctuation state, allowing the population to converge rapidly and precisely to the globally optimal 4-channel combination, with classification performance achieving significant improvement.

**Table 8:** Comparison of WOA, GWO, and the proposed WOA-GWO hybrid algorithm.

Comparison dimension	WOA	GWO	WOA-GWO
Biological inspiration	Humpback whale bubble-net feeding behavior	Grey wolf hierarchy and hunting behavior	Integration of both biological mechanism advantages
Core search mechanism	Spiral position updating, encircling prey mechanism, random search operator	α/β/δ/ω hierarchical structure, three-wolf guidance mechanism, encircling strategy	WOA spiral and GWO hierarchical structure with nonlinear convergence factor, chaotic initialization
Exploration-exploitation balance	Strong global exploration capability, but prone to local optima in later stages	Strong local exploitation capability, but insufficient global search	Dynamic balance: rapid exploration in early stage (0–100 iterations), smooth transition in middle stage (100–200 iterations), stable convergence in later stage (200–300 iterations)
Key parameters	Population size, maximum iterations, coefficient vectors A/C, probability parameter p	Population size, maximum iterations, convergence factor a, coefficient vectors A/C	Incorporates all parameters from WOA and GWO, requires coordinated fusion weights
Population diversity characteristics	Conservative exploration (0.36–0.48), slow convergence, prone to local optima	Excessive exploration (reaches high values initially), difficult convergence, resource waste	Three-stage dynamic equilibrium: early stage 0.32–0.47, middle stage 0.37–0.43, later stage 0.35–0.40
Time complexity	Lower	Moderate	Higher, but with significant accuracy improvement
Improvements in this study	–	–	Chaotic map initialization for enhanced diversity, nonlinear convergence factor for dynamic adjustment, random perturbation to avoid local stagnation, ReliefF feature fusion for dimensionality reduction

**Figure 15:**
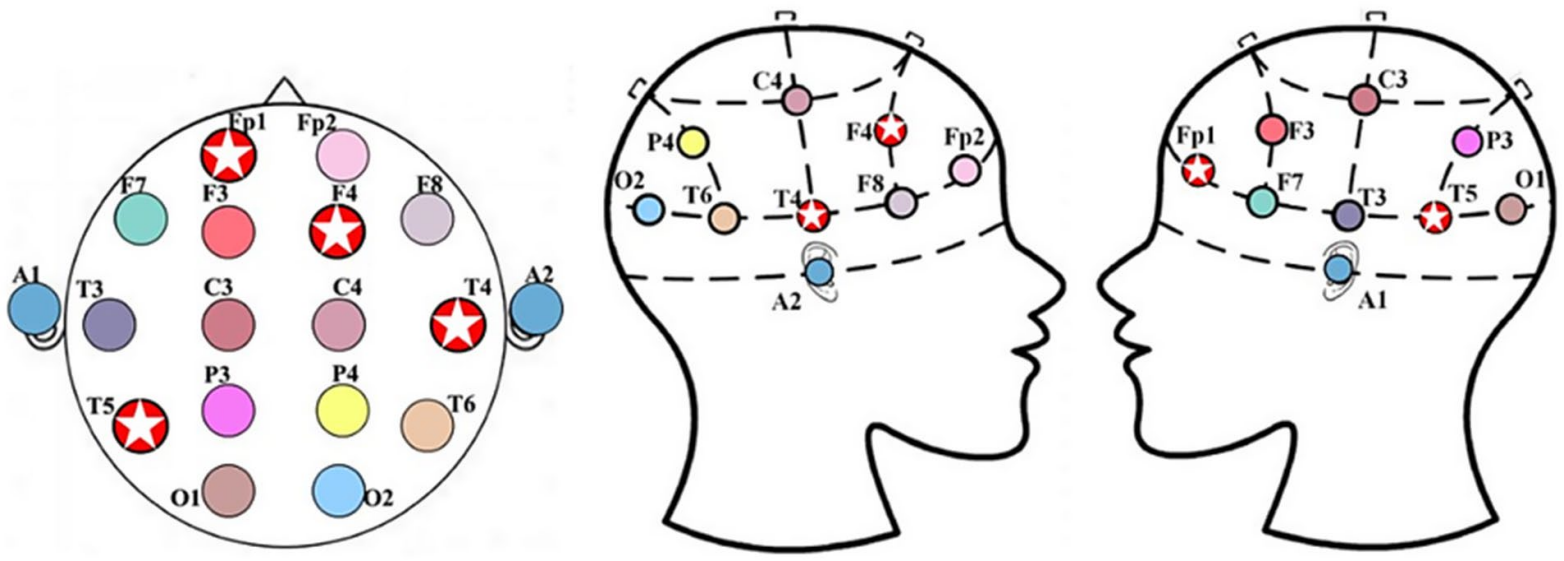
Visualization maps of the four channels (Fp1, F4, T4, T5) selected by WOA-GWO.

According to the international 10–20 system, the four core electrode channels (T5, FP1, T4, F4) identified in this study precisely cover the key brain regions affected during the pathological progression of AD, providing neurophysiological interpretability for the model’s high diagnostic performance (Figure 15): Channel T5 corresponds to the posterior left temporal lobe, adjacent to medial temporal structures such as the hippocampus and entorhinal cortex—the core initial region where β-amyloid deposition and neurofibrillary tangles first occur in AD, and the neurodegenerative changes in this area directly trigger abnormal patterns of local electroencephalographic activity, making it a critical window for capturing early AD pathophysiological signals; Channel T4 is located in the anterior right temporal lobe, which is primarily involved in linguistic semantic processing and emotional memory integration, representing a typical affected region in the mid-stage pathological spread of AD; its functional impairment is closely associated with common AD symptoms such as anomia and semantic memory decline, and the specific changes in its electroencephalographic activity provide a sensitive feature basis for the model to distinguish AD from CN individuals; Channel F4 corresponds to the middle-inferior right frontal lobe, a core brain region for executive function regulation that exhibits functional connectivity reorganization in the early stage of AD; the neuronal firing rhythm disturbances caused by β-amyloid deposition and the compensatory neuroplasticity of subcortical–cortical circuits collectively shape the unique electroencephalographic activity pattern of this region, which is also the key reason for the algorithm to prioritize it as a core channel; Channel FP1 is located in the left frontopolar region, a key node of the default mode network (DMN) involved in high-order cognitive control and episodic memory retrieval; early AD-related DMN dysfunction leads to significant low-frequency oscillatory abnormalities in this area, whose neural activity changes often precede obvious clinical symptoms, thus offering a highly sensitive physiological indicator for detecting early AD pathology at the network level. The combination of these four channels not only covers the medial temporal lobe where AD pathology originates but also includes the frontal lobe and DMN-related regions involved in early functional compensation, aligning with the spatiotemporal pattern of AD pathological spread and verifying the effectiveness of the WOA-GWO algorithm in selecting highly discriminative and interpretable EEG channels, thereby providing a neurophysiological basis for the clinical translation of lightweight early AD diagnostic systems.

The four selected channels demonstrate not only high discriminability at the computational level but also systematically cover the typical pathological trajectory of AD at the neurobiological level, extending from the medial temporal lobe initiation to bilateral temporal lobe propagation and subsequent functional involvement of the frontal lobe (particularly the frontopolar and middle-inferior frontal regions). The fused features of SAV, STD, and STA transcend the limitations of “purely data-driven” channel selection, endowing the selected channels with explicit physiopathological significance, which fully validates the pathological interpretability and mechanistic consistency of the proposed method in channel selection. The core advantage lies in establishing a precise matching between “pathology-related features and highly discriminative channels” through the WOA-GWO algorithm, grounded in the physiological relevance of SODP time-domain features. The channel set selected by this algorithm effectively covers the core brain regions affected by AD pathology, capturing functional differences between AD and CN individuals across three dimensions (amplitude, fluctuation, and energy-thereby) providing critical feature support for high-precision automated diagnosis.

In the technical spectrum of intelligent Alzheimer’s disease detection, the WOA-GWO optimization framework proposed in this study exhibits significant complementarity with deep learning models such as Convolutional Neural Networks (CNN), Long Short-Term Memory networks (LSTM), and Transformers in terms of functional positioning. Deep learning architectures typically serve as back-end classifiers, relying on large-scale datasets (often requiring thousands of cases) to drive end-to-end automatic feature learning and complex spatiotemporal pattern recognition. In contrast, the present method is positioned as a front-end optimization module, specializing in sparse channel selection and feature dimensionality reduction in high-dimensional EEG spaces. This distinction highlights the clinical translational advantages of our framework across three critical dimensions. First, regarding small-sample adaptability, WOA-GWO achieves stable convergence and reliable channel optimization without requiring massive annotated data, effectively overcoming the overfitting bottleneck arising from the scarcity of high-quality clinical EEG cohorts. Second, in terms of physiological interpretability, unlike the “black-box” nature of deep learning, the 4-channel subset explicitly selected by this algorithm systematically covers the typical pathological progression trajectory of AD from medial temporal lobe initiation to frontal lobe involvement. Combined with SODP time-domain features encompassing amplitude, fluctuation, and energy dimensions, this provides traceable neurophysiological evidence for diagnostic decisions (Cacciotti et al., 2024; Ghassemkhani et al., 2025). Third, concerning computational efficiency and deployment feasibility, this framework operates efficiently in standard CPU environments and streamlines electrode configuration by 75%, significantly reducing hardware power consumption and subject preparation burden, better aligning with the practical requirements of portable EEG devices and grassroots screening scenarios (Suárez-Marcote et al., 2026). This method is not intended to replace deep learning paradigms, but rather focuses on front-end optimal subset search for features and channels followed by classification using lightweight classifiers. In future research, we will explore hybrid architectures that combine hybrid intelligent optimization algorithms with deep learning classifiers for final prediction, thereby synergistically fusing the optimization-driven sparsity of the former with the data-driven representational capacity of the latter.

To maximally mitigate overfitting risks in small-sample scenarios and ensure model generalization capability, this study employed a dual evaluation strategy combining LOSO cross-validation with 5-fold cross-validation. Simultaneously, by leveraging the WOA-GWO hybrid optimization algorithm for substantial channel dimension compression and ReliefF feature fusion dimensionality reduction, we systematically circumvented data sparsity and model complexity inflation issues in high-dimensional spaces at the algorithmic mechanism level. ReliefF serves not as an optional “performance tuning module” in this framework, but as a critical bridge connecting high-dimensional nonlinear feature spaces with combinatorial optimization algorithms. Direct input of raw SODP geometric features and entropy features into WOA-GWO would induce severe “curse of dimensionality” effects, leading to exponential search space explosion, convergence stagnation, or even computational overflow. As substantiated by Srivastava and Tayal (2024) regarding stability in high-dimensional data and the feature weight fusion architecture proposed by Wang and Zhao (2025), adaptive feature weighting and dimensionality reduction constitute prerequisite conditions for ensuring effective search in metaheuristic algorithms. In this study, ReliefF compresses redundant high-dimensional feature spaces into compact, efficiently searchable subspaces by quantifying feature discriminative power and allocating adaptive weights, thereby enabling WOA-GWO to precisely locate globally optimal channel subsets. This design logic aligns highly with Mortatha and Abdulah (2025) regarding the guiding role of feature preprocessing for metaheuristic algorithms, further validating the methodological rationality and engineering feasibility of the hierarchical “dimensionality reduction first, optimization follow-up” strategy in addressing small-sample high-dimensional EEG feature selection problems.

Although this study has achieved preliminary validation in terms of algorithm design and feature engineering, its scope of application and boundaries for generalization must be objectively evaluated. The current model was built on a single-center cohort with a relatively limited sample size, which may to some extent compromise the model’s generalization ability in heterogeneous populations. Nevertheless, such a sample size is typical in early exploratory studies of EEG-based biomarkers for Alzheimer’s disease, mainly due to the requirements for precisely annotated clinical data under strict inclusion criteria and the resource constraints of high-quality resting-state EEG acquisition. Notably, the core channels screened in this study are not purely data-driven outcomes; they are highly consistent with the known neuropathological progression of Alzheimer’s disease. This pathophysiological coherence provides biological prior support for cross-cohort migration of the model, effectively mitigating the risk of data distribution shift potentially caused by single-center acquisition. Standardized public benchmarks are crucial for horizontal algorithm comparison and reproducibility verification. However, since existing public EEG databases lack AD resting-state EEG cohorts that are strictly labeled under unified clinical diagnostic criteria and feature highly consistent acquisition protocols and electrode impedance control, this study prioritized a single-center high-quality clinical cohort to ensure the signal-to-noise ratio of feature extraction and label reliability.

In future research, external validation will be placed at the core: on the one hand, active connections will be made with OpenNeuro, the extended module of ADNI-EEG, and multi-center EEG collaborative networks to conduct generalization performance assessments across devices, populations, and acquisition environments (Li et al., 2026); on the other hand, domain adaptation and transfer learning strategies are planned to be introduced to further reduce signal distribution differences across clinical centers. Future work will systematically promote large-scale prospective multi-center validation, integrate hybrid frameworks of intelligent optimization algorithms and deep learning (Amrir et al., 2026), and incorporate multi-band EEG dynamic features, structural/functional imaging, and blood biomarkers to build an interpretable multi-modal diagnostic framework. Through continuous external benchmark testing and clinical cohort expansion, the lightweight channel optimization paradigm proposed in this study is expected to provide a more universal and evidence-based technical pathway for early screening of Alzheimer’s disease and portable brain–computer interface devices.

## 6 Conclusion

This study proposes a novel framework combining nonlinear dynamic feature fusion and multi-strategy enhanced WOA-GWO optimization for AD detection with sparse EEG channels. The experimental results show that the framework achieves a classification accuracy of 96.97% with four optimal EEG channels (T5, FP1, T4, F4), and the WOA-GWO algorithm outperforms the original WOA and GWO in convergence and optimization performance. The selected channels and features have clear neurophysiological interpretability for AD pathology, providing evidence for AD electrophysiological biomarkers. This work offers a reliable computational framework for the development of lightweight, portable AD diagnostic systems, and has great potential for clinical translation in early AD screening and progression monitoring.

## Data Availability

The data analyzed in this study is subject to the following licenses/restrictions: the raw EEG data generated in this study are not publicly available due to privacy and ethical restrictions, as the data contain sensitive clinical information of Alzheimer’s disease patients and healthy elderly volunteers. Access to the data can be granted only upon reasonable request from the corresponding author, with prior approval from the Scientific Research Ethics Committee of Tianjin University of Technology and Education, in compliance with local data protection regulations. Requests to access these datasets should be directed to yqche@tju.edu.cn.
